# Joint polygenic and environmental risks for childhood attention‐deficit/hyperactivity disorder (ADHD) and ADHD symptom dimensions

**DOI:** 10.1002/jcv2.12152

**Published:** 2023-03-16

**Authors:** Michael A. Mooney, Peter Ryabinin, Hannah Morton, Katharine Selah, Rose Gonoud, Michael Kozlowski, Elizabeth Nousen, Jessica Tipsord, Dylan Antovich, Joel Schwartz, Megan M. Herting, Stephen V. Faraone, Joel T. Nigg

**Affiliations:** ^1^ Division of Bioinformatics and Computational Biology Department of Medical Informatics and Clinical Epidemiology Oregon Health & Science University Portland Oregon USA; ^2^ Knight Cancer Institute Oregon Health & Science University Portland Oregon USA; ^3^ Department of Psychiatry Center for ADHD Research Oregon Health & Science University Portland Oregon USA; ^4^ Department of Environmental Health Harvard T.H. Chan School of Public Health Boston Massachusetts USA; ^5^ Department of Population and Public Health Sciences Keck School of Medicine of the University of Southern California Los Angeles California USA; ^6^ Department of Pediatrics Children's Hospital Los Angeles Los Angeles California USA; ^7^ Department of Psychiatry SUNY Upstate Medical University Syracuse New York USA; ^8^ Department of Behavioral Neuroscience Oregon Health & Science University Portland Oregon USA

**Keywords:** ADHD, environment, gene‐environment interplay, geocoding, polygenic scores

## Abstract

**Background:**

attention‐deficit/hyperactivity disorder (ADHD) is associated with both polygenic liability and environmental exposures, both intrinsic to the family, such as family conflict, and extrinsic, such as air pollution. However, much less is known about the interplay between environmental and genetic risks relevant to ADHD—a better understanding of which could inform both mechanistic models and clinical prediction algorithms.

**Methods:**

Two independent data sets, the population‐based Adolescent Brain Cognitive Development Study (ABCD) (*N* = 11,876) and the case‐control Oregon‐ADHD‐1000 (*N* = 1449), were used to examine additive (G + E) and interactive (GxE) effects of selected polygenic risk scores (PRS) and environmental factors in a cross‐sectional design. Genetic risk was measured using PRS for nine mental health disorders/traits. Exposures included family income, family conflict/negative sentiment, and geocoded measures of area deprivation, lead exposure risk, and air pollution exposure (nitrogen dioxide and fine particulate matter).

**Results:**

ADHD PRS and family conflict jointly predicted concurrent ADHD symptoms in both cohorts. Additive‐effects models, including both genetic and environmental factors, explained significantly more variation in symptoms than any individual factor alone (joint *R* ^2^ = .091 for total symptoms in ABCD; joint *R* ^2^ = .173 in Oregon‐ADHD‐1000; all delta‐*R* ^2^ *p*‐values <2e‐7). Significant effect size heterogeneity across ancestry groups was observed for genetic and environmental factors (e.g., *Q* = 9.01, *p* = .011 for major depressive disorder PRS; *Q* = 13.34, *p* = .001 for area deprivation). GxE interactions observed in the full ABCD cohort suggested stronger environmental effects when genetic risk is low, though they did not replicate.

**Conclusions:**

Reproducible additive effects of PRS and family environment on ADHD symptoms were found, but GxE interaction effects were not replicated and appeared confounded by ancestry. Results highlight the potential value of combining exposures and PRS in clinical prediction algorithms. The observed differences in risks across ancestry groups warrant further study to avoid health care disparities.

**Key points**

- Both attention‐deficit/hyperactivity disorder (ADHD) polygenic risk score (PRS) and family environment were robustly associated with ADHD symptoms in two independent cohorts.
- Additive effects of PRS and environment explained significantly more variation in symptoms than either domain alone, indicating the potential utility of combining risk factors in clinical prediction algorithms.
- Genotype‐by‐environment interaction interactions observed were confounded by ancestry and did not replicate, suggesting these effects are likely small for the PRS and exposures examined here.
- Differences in effects of PRS and exposures across ancestry groups warrants further research to avoid health care disparities.

## INTRODUCTION

It has long been known that attention‐deficit/hyperactivity disorder (ADHD) is associated with social disadvantage (Russell et al., 2016), even after adjusting for co‐occurring disruptive behavior problems (Miller et al., 2018). More recently, evidence has emerged of a possible role for other exposures. Notable is the limited study of potential effects of pollution measures, especially in a genotype‐by‐environment interaction (GxE) context. Yet these are important for their public health significance, ubiquity, and ease of standardized evaluation by geospatial coding. Here, we examine lead exposure risk (Moore et al., 2022; Nigg et al., 2016) and measures of air pollution exposure: nitrogen dioxide (NO_2_) and fine particulate matter (PM2.5) (Donzelli et al., 2019; Thygesen et al., 2020). Mixed results from previous studies highlight the need for further investigation of the association of these air pollutants with conditions such as ADHD (Zhang et al., 2022). These exposures can be considered extrinsic to the family environment.

Another set of relevant environments are intrinsic to the family. While family income is one obvious risk related to low social economic status, others pertain. Most commonly noted are features of the family emotional climate, such as conflict, criticism, and negative tone/sentiment (Harold et al., 2013; Musser et al., 2016; Peris & Hinshaw, 2003; Richards et al., 2014).

How and to what extent these intrinsic and extrinsic risks intersect with ADHD's well‐established genetic liability is of substantial importance but remains poorly described. Clarification of the joint contributions of exposure and polygenic measures can inform both mechanistic understanding and potential clinical prediction algorithms.

The literature on GxE and ADHD has focused on candidate gene variants that are not captured in genome wide association studies (GWAS), such as the DRD4 and MAOA variable tandem repeats (Kanarik et al., 2022), along with intrinsic risk factors (e.g., parenting). The opportunity provided by GWAS studies to further evaluate additive and interactive models is substantial and warrants broader investigation.

A recent large‐scale genome‐wide association study (Demontis et al., 2019) underscored the highly polygenic nature of ADHD. Numerous subsequent studies have demonstrated that a polygenic risk score (PRS), representing the cumulative risk of common single nucleotide polymorphisms across the genome, significantly predicts ADHD diagnosis, and is significantly correlated with ADHD symptoms and related traits in the general population (Demontis et al., 2019; Green et al., 2022; Ronald et al., 2021; Taylor et al., 2019), as well as associated features such as executive functioning and emotional dysregulation (Nigg et al., 2018, 2020).

However, the fact that many psychiatric disorders share genetic risk factors, previously known from behavioral genetic studies, has also been demonstrated recently using GWAS data. The significant genetic correlation among many neurodevelopmental and mental health conditions suggests that PRS are not entirely specific to individual disorders, and that combining information from multiple PRS may improve predictive models (Barnett et al., 2022; Neumann et al., 2022; Waszczuk et al., 2021).

Studies using whole‐genome PRS derived from GWAS in a GxE context are still relatively new (Palladino et al., 2019). Yet, because PRS explain significantly more trait variation than individual genetic variants, they are becoming an important approach for investigating the interplay between genetic and environmental risk factors for complex diseases, including mental health‐related traits (Agnew‐Blais et al., 2022; He & Li, 2022; Stojanovski et al., 2019, Øtergaard et al., 2020). Nevertheless, robust evidence of interactions between PRS and environmental exposures for ADHD and other disorders has so far been elusive (He and Li, 2022, Øtergaard et al., 2020).

Here, we take advantage of increasingly informative PRS, the growing availability of geospatial coding of environmental risk factors to standardize assessment in large samples, and the large‐scale American Adolescent Brain Cognitive Development℠ study (ABCD Study®) of over 11,000 children selected from around the nation, along with the unique Oregon‐ADHD‐1000 case‐control sample (Nigg et al., 2023). Examination of results across both population and case‐control (community recruited) samples can clarify both public health and clinically relevant effects, as well as the reproducibility of findings.

In the present study we aimed to test the following hypotheses: (1) that multiple genetic (PRS) and environmental risk factors are additively associated with both dimensional measures and ADHD/non‐ADHD status, and (2) that genetic effects are dependent upon environmental context (or vice versa). Novel features of this report include the use of multiple PRS, geocoded exposure variables, and the inclusion of risk factors both intrinsic and extrinsic to the family in a GxE study of ADHD. Moreover, risk‐inducing environmental exposures are more heavily concentrated among minoritized racial and ethnic groups in the U.S. (Liu et al., 2021; Mullen et al., 2020), and differential rates of identification and treatment of ADHD across racial and ethnic groups remains a complex challenge for understanding population needs, risks, and outcomes in ADHD (Chung et al., 2019; Yang et al., 2022). Yet studies of polygenic scores in non‐European ancestry populations are rare, leaving a critical gap in knowledge. Therefore, despite some power limitations, we also include ancestry‐stratified analyses here to begin to supply the necessary data for future reviews.

## MATERIALS AND METHODS

### Participants

All analyses were done in two independent cohorts, the ABCD Study cohort (*N* = 11,876) and the Oregon‐ADHD‐1000 (*N* = 1449). The ABCD cohort is a large, 21‐site, diverse sample of children aged 9–10 years at baseline (Jernigan et al., 2018; Volkow et al., 2018), which is publicly available on the NIMH Data Archive (http://dx.doi.org/10.15154/1523041) (Barch et al., 2018; Uban et al., 2018). The children have been genotyped and are followed with extensive behavioral, cognitive, clinical, and MRI measures annually. Baseline data were examined here. Dimensional measures of ADHD symptoms were studied and a matched case‐control subsample was created as explained in Supporting Information S1.

The Oregon‐ADHD‐1000 is a community recruited case‐control cohort of youth age 7–11 years (Karalunas et al., 2017; Mooney, Bhatt et al., 2020; Mooney, Ryabinin, et al., 2020; Nigg et al., 2018, 2020) followed longitudinally for 12 years. Baseline data were examined here. Human subject protection and ethics approval were obtained from the local University Institutional Review Board. A parent/legal guardian provided written informed consent, and children provided written assent.

### ADHD assessments

In ABCD, total ADHD symptoms were measured using the Child Behavior Checklist (CBCL) ADHD DSM‐oriented scale (Achenbach & Rescorla, 2001). In the Oregon‐ADHD‐1000, total ADHD symptoms were measured using the parent‐reported ADHD Rating Scale (ADHDRS) (DuPaul et al., 1998). Norm‐referenced *T*‐scores were used for both symptom measures. A sensitivity analysis examining the consistency of polygenic and environmental effects on additional measures of ADHD symptoms, and on inattention and hyperactivity/impulsivity symptoms separately, is described in Supporting Information S1.

In the ABCD cohort, ADHD/non‐ADHD status was based on the Tier 4 criteria described by Cordova et al. (2022), but was expanded to include participants who met diagnostic criteria in the past and still had elevated symptoms, as explained in the supplement to (Cordova et al., 2022) (details in Supporting Information S1). In the Oregon‐ADHD‐1000, ADHD diagnosis was performed using a multi‐method, multi‐informant, multi‐reviewer best‐estimate protocol (details in Supporting Information S1). The full diagnostic assessment procedure has been described previously (Nigg et al., 2018).

### Polygenic risk scores

Genome‐wide genotype data were available for both cohorts, and details about data collection and processing have been published previously (Cordova et al., 2022; Nigg et al., 2018). Using imputed genotypes based on the 1000 Genomes reference panel (phase 3), PRS for ADHD, major depressive disorder (MDD), autism spectrum disorder (ASD), bipolar disorder (BP), schizophrenia (SCZ), anxiety (ANX), cannabis use disorder (CUD), alcohol use disorder (AUDIT), and alcohol dependency (ALCDEP) were calculated for each cohort. Details about the discovery data sets used for each PRS are included in Table S1. The LDpred method (Vilhjálmsson et al., 2015) was used to calculate each PRS. All unrelated, European‐ancestry ABCD participants were used to estimate linkage disequilibrium, given that the discovery genome‐wide association studies were done in primarily European‐ancestry samples. Given the known highly polygenic nature of the traits studied here (Demontis et al., 2023), the proportion of causal‐variants was set to 0.3.

### Identification of ancestry groups

To examine whether effects generalize across different ancestry groups, we performed stratified analyses for the three ancestry groups reasonably well‐represented in ABCD: White/European, Hispanic/Latino, and Black/African‐American. The ancestry groups were defined using a combination of genetic data and self‐reported race/ethnicity data (details in Figure S1). For the Oregon‐ADHD‐1000, only a European‐ancestry subgroup was defined, given the small number of participants with Hispanic/Latino or Black/African‐American ancestry.

### Environmental exposure measures

#### Extrinsic geocoded indices

The following risk factors were evaluated by geo‐spatial coding of the participants' residential addresses: Area deprivation index (ADI), lead exposure risk, and levels of NO_2_ and fine particulate matter (PM2.5). Geospatial location data were linked to external reference databases using methods described previously (Fan et al., 2021). ADI was calculated as a weighted sum of 17 factors from the American Community Survey data (Singh, 2003). Lead exposure risk was based on the Washington State Department of Health Childhood Lead Risk map. Each participant was assigned to a decile of risk relative to risk levels across the country (Frostenson, 2016). Air pollution concentrations (PM2.5 and NO_2_) were estimated using hybrid spatiotemporal models, which utilize satellite‐based aerosol optical depth models, land‐use regression, and chemical transport models (Di et al., 2019, 2020). In ABCD, the average was calculated over the calendar year 2016, which corresponded with the initial enrollment period for the baseline study visit. In the Oregon‐ADHD‐1000 cohort, the average was calculated over the calendar year of, or preceding (for those visits before May 1), the baseline study visit date (between 2009 and 2012).

#### Intrinsic family risk factors

Family income and family sentiment/conflict were evaluated to index the proximal family environment. Family income scales for both cohorts are described in Supporting Information S1.

In ABCD, family conflict was measured with the family conflict subscale of the widely‐used Family Environment Scale (Moos & Moos, 1994). In the Oregon‐ADHD‐1000 cohort, parental expressed emotion and implicit sentiment were assessed via the Five Minute Speech Sample (FMSS) (Magaña et al., 1986). To obtain a single measure of sentiment/emotional tone for each FMSS transcript, pre‐trained computerized text classification models were applied to each sentence in the transcript, producing a probability that the sentence contains negative sentiment (details in Supporting Information S1). The probability of negative sentiment in each sentence was averaged across all sentences in the transcript to obtain a mean negative sentiment score (Selah et al., 2022).

### Statistical analyses

Missing data in each cohort was handled using multiple imputation as implemented in the *mice* R package (van Buuren and Groothuis‐Oudshoorn, 2011) (additional details provided in Tables S2 and S3). Effect estimates were determined by pooling estimates across 50 imputed data sets using the pool function in the *mice* package. For all models, outcomes (dependent variables) and predictors of interest were standardized (mean = 0, standard deviation = 1) and the standardized regression coefficients reported. Sensitivity analyses were performed to determine if variable transformation affected the observed associations, with no appreciable effect on results (see Supporting Information S1).

Main effects of each PRS and each environmental exposure on total ADHD symptoms were examined in all three cohorts (ABCD, ABCD matched case‐control subsample, and Oregon‐ADHD‐1000), while effects on the categorical outcome (ADHD/non‐ADHD status) was examined only in the two case‐control cohorts. Models were fit using the *geepack* R package (Højsgaard et al., 2006), specifying an exchangeable correlation structure, and clustering on family ID to handle sibling relatedness (see Table S4). Covariates were included for age, sex, site (for ABCD only) and 10 genomic principal components. Linear models were fit for the dimensional symptom measures, while logistic models were fit for ADHD/non‐ADHD status. Additive effects were examined by fitting a “full additive‐effects” model that included all genomic and exposure variables. Backwards selection was performed to identify a reduced additive‐effects model containing only corrected significant (*p*‐values <.00333; see below) genomic and/or exposure variables. GxE effects were examined with models that included interaction terms for each pair of genomic and exposure variables—all pairs tested individually in separate models. For each model, a pooled R‐squared value was calculated as the mean of the *R* ^2^ values across all 50 imputations (van Ginkel, 2019). A pooled *F*‐test, using Rubin's rules to combine test statistics across imputations, was used to test the significance of the difference in *R* ^2^ values between two nested models following recommendations (van Ginkel, 2019). For the logistic models, the mean area under the receiver operating curve across the 50 imputations is reported.

A discovery/replication framework was utilized, with the larger ABCD cohort acting as the discovery data set, and the Oregon‐ADHD‐1000 as the replication data set. Primary analyses of dimensional measures were done separately in each ancestry subgroup (defined above) within each cohort. In ABCD, results from each of the three ancestry subgroups were compared and meta‐analyzed, using a random‐effects meta‐analysis implemented with the *meta* R package (Balduzzi et al., 2019). A sensitivity analyses examining the impact of covarying genomic PCs calculated across the full ABCD cohort versus PCs calculated within each ancestry subgroup separately found no meaningful differences in effect estimates (see Supporting Information S1).

The categorical outcome (ADHD/non‐ADHD status) was analyzed only within the European‐ancestry subgroups, given the small number of cases in the other ancestry groups. Additionally, analyses for all outcomes were also conducted in each full cohort (all participants regardless of ancestry).

Due to the large number of PRS and exposures examined, a Bonferroni multiple testing correction was applied separately to the set of models examining individual main effects (*N* = 15 predictors tested) and the set of models examining interaction effects (*N* = 54 G‐E pairs tested). Individual main effects with *p* < .00333 and interactions with *p* < 9.26e‐4 were considered statistically significant in discovery analyses in ABCD. To assess replication in the Oregon‐ADHD‐1000, a significance threshold of *p* < .05 was used. The Supporting Information S1 provides effect sizes for non‐significant GxE findings at *p* < .10.

## RESULTS

### Overview of cohorts

The distribution of ADHD symptoms, ADHD diagnoses, environmental exposures, and demographics for all three cohorts are reported in Table 1. PRS and exposure distributions are reported in Figures S2 and S3. Given the different study ascertainment methods, there are significantly different proportions of ADHD diagnoses in the ABCD and Oregon‐ADHD‐1000 cohorts. As a result of the case‐control design, mean symptoms are higher and the proportion of females is lower in the Oregon‐ADHD‐1000 cohort (*p*‐values <4e‐10). In terms of family environment and exposures, the Oregon‐ADHD‐1000 had significantly lower median family income, ADI, lead risk, and NO_2_ exposure, but significantly higher PM2.5 exposure (all *p*‐values <.002). The same patterns hold when comparing the Oregon‐ADHD‐1000 to the ABCD matched case‐control sub‐sample (all *p*‐values <.003). Likewise, results were the same when comparing the European‐ancestry subgroups of the two cohorts, except there was no significant difference in lead exposure risk.

**TABLE 1:** Overview of cohorts.

	ABCD	ABCD matched case‐control	Oregon‐ADHD‐1000
Total *N*	11,867	1620	1449
Age (years)	9.9 (0.6)	9.9 (0.6)	9.4 (1.6)
% Female	47.8%*	31.3%*	39.1%
*N* with genotypes	10,497	1427	770
% White, European	46.9% (*N* = 5562)	45% (*N* = 732)	42.0% (*N* = 609)
% Hispanic/Latino	9.4% (*N* = 1110)	NA	NA
% Black/African‐American	12.0% (*N* = 1423)	NA	NA
% ADHD cases	4.5% (*N* = 540)*	33.3% (*N* = 540)*	49.8% (*N* = 721; missing = 31)
Total ADHD symptoms *T*‐score	53.2 (5.6)*	56.4 (8.8)*	61.5 (16.4)
Family income	Median = $75,000–$99,999*	Median = $75,000–$99,999*	Median = $50,000–$74,999
Family conflict	2.54 (2.0)	2.71 (2.0)	NA
Negative sentiment	NA	NA	0 (0.95)
Lead risk	5.1 (3.1)*	5.00 (3.1)	4.36 (2.9)
NO_2_ exposure	18.6 (5.8)*	18.5 (5.7)*	17.4 (2.4)
PM2.5 exposure	7.7 (1.6)*	7.6 (1.4)*	7.8 (1.4)
ADI	94.7 (21.1)*	94.8 (21.8)*	90.5 (12.8)

*Note*: The mean and standard deviation (in parentheses) are reported unless otherwise specified. An asterisk (*) indicates a significant difference in distribution compared to the Oregon‐ADHD‐1000.

Abbreviations: ABCD, Adolescent Brain Cognitive Development Study; ADHD, attention‐deficit/hyperactivity disorder; ADI, area deprivation index.

### Extent of gene‐environment correlation

Small but statistically reliable correlations were observed among the various PRS, as expected given the known genetic correlation among psychiatric disorders. In the European‐ancestry subgroups of both cohorts, the ADHD PRS was most strongly correlated with PRS for ASD (*r* = .29 and .28 for ABCD and Oregon‐ADHD‐1000, respectively), MDD (*r* = .23 and .17) and CUD (*r* = .17 and .21) (all *p*‐values <4e‐5).

Significant correlations between PRS and environmental measures were also seen in both cohorts. In ABCD the ADHD PRS was weakly, but significantly, correlated with ADI (*r* = .088, *p* = 1.9e‐10), family income (*r* = −.12, *p* < 2e‐16), family conflict (*r* = .058, *p* = 1.40e‐5), and NO_2_ levels (*r* = −.056, *p* = 3.81e‐5). In the Oregon‐ADHD‐1000 cohort, ADHD PRS was correlated with negative sentiment (*r* = .12, *p* = 5.46e‐3) and lead risk (*r* = −.083, *p* = .0421).

**FIGURE 1:**
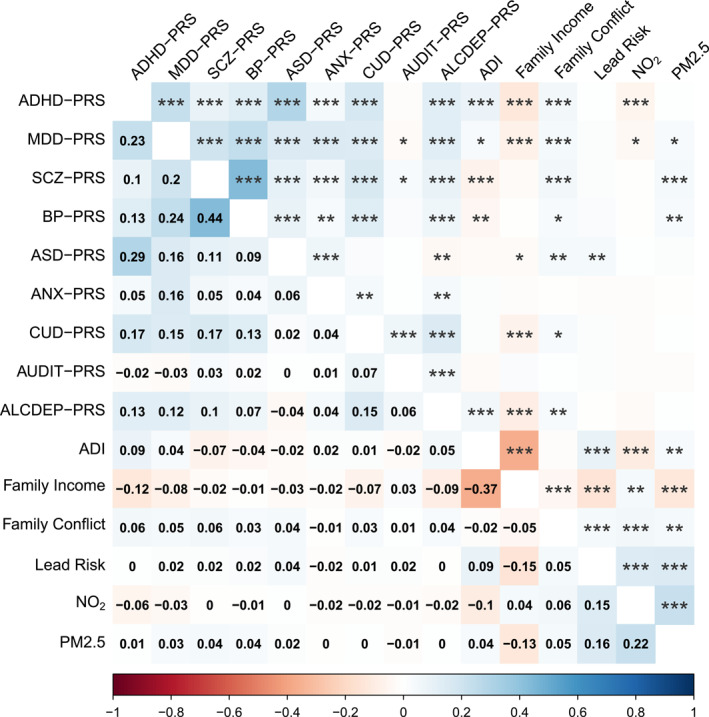
Correlation matrix of PRS and exposures for the European‐ancestry subgroup of the ABCD cohort. Correlation coefficients are shown in the lower triangle and significance codes in the upper triangle, with *, **, and *** representing *p* < .05, *p* < .01, and *p* < .001, respectively. ABCD, Adolescent Brain Cognitive Development Study; PRS, polygenic risk score.

Correlations among environmental exposures were small to moderate (max *r* = −.37, for ADI and family income), indicating the measures represent distinct risks. Correlations among all PRS and environmental measures in the European‐ancestry subgroups are reported in Figure 1 and Figure S4.

### Main effects of exposures and PRS

**FIGURE 2:**
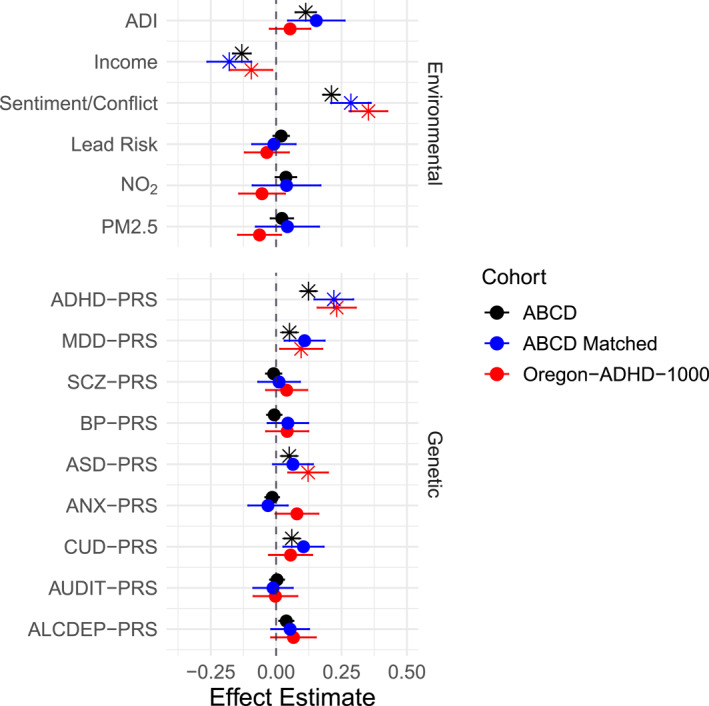
Univariate main effects (standardized regression coefficient) of all exposures and PRS on total ADHD symptoms for the European‐ancestry subsamples in each cohort. Point estimates indicated with an asterisk (*) are either (a) statistically significant after multiple‐testing correction in ABCD, or (b) significantly replicated in the Oregon‐ADHD‐1000 (*p* < .05). Total ADHD symptoms were measured with the CBCL ADHD DSM‐oriented scale in ABCD, and the ADHD Rating Scale in the Oregon‐ADHD‐1000 cohort. ABCD, Adolescent Brain Cognitive Development Study; ADHD, attention‐deficit/hyperactivity disorder; CBCL, Child Behavior Checklist; PRS, polygenic risk score.

Main effects for all PRS and environmental exposures on total ADHD symptoms are presented for the European‐ancestry subgroups of each cohort in Figure 2, with family conflict/negative emotional tone and, unsurprisingly, the ADHD PRS showing the strongest and most consistent effects.

For dimensional analysis of total ADHD symptoms, in the ABCD European‐ancestry subgroup, ADI ($\hat{\beta }$ = .113, *p* = 1.45e‐8), family income ($\hat{\beta }$ = −.131, *p* = 4.75e‐14), and family conflict ($\hat{\beta }$ = .211, *p* < 2e‐16) were all significantly associated with total ADHD symptoms in the expected direction. PRS for ADHD ($\hat{\beta }$ = .124, *p* < 2e‐16), MDD ($\hat{\beta }$ = .051, *p* = 1.1e‐4), ASD ($\hat{\beta }$ = .050, *p* = 3.1e‐4), and CUD ($\hat{\beta }$ = .060, *p* = 2.37e‐5) were also significantly associated with total symptoms.

Replication in the Oregon‐ADHD‐1000 European‐ancestry subgroup was observed for effects of familial negative sentiment ($\hat{\beta }$ = .353, *p* < 2e‐16), family income ($\hat{\beta }$ = −.095, *p* = .0208), ADHD PRS ($\hat{\beta }$ = .232, *p* = 1.0e‐9), MDD PRS ($\hat{\beta }$ = .096, *p* = .0206), and ASD PRS ($\hat{\beta }$ = .122, *p* = .0017) on total ADHD symptoms.

Results for all ancestry groups in ABCD are shown in Figure S5, suggesting important differences in risk factors. For example, ADI and family income were significantly associated with total symptoms in only the European‐ancestry subgroup, while family conflict was strongly associated in all subgroups. Furthermore, none of the PRS were associated with outcomes in the Black/African‐American subgroup. It should be noted that effect sizes were smaller in the Black/African‐American subgroup for all exposures and PRS that were significant in other subgroups, indicating the lack of association is not simply due to the smaller sample size (i.e., larger confidence intervals). Meta‐analysis of effects across the three ancestry groups in ABCD confirmed significant associations between total ADHD symptoms and family income ($\hat{\beta }$ = −.091, *p* = .001), family conflict ($\hat{\beta }$ = .204, *p* < 2e‐16), and ADHD‐PRS ($\hat{\beta }$ = .107, *p* = 1.32e‐5). However, tests of heterogeneity suggest real differences in the effects of risk factors across the three ancestry groups in ABCD for ADI (*Q* = 13.34, *p* = .00127) and income (*Q* = 6.83, *p* = .0329), as well as PRS for ADHD (*Q* = 5.68, *p* = .0583), MDD (*Q* = 9.01, *p* = .0110), and ASD (*Q* = 13.3, *p* = .00130).

For the categorical analysis of ADHD diagnosis, income (log(OR) = −.322, *p* = 7.93e‐5), family conflict (log(OR) = .517, *p* = 2.02e‐9), and ADHD‐PRS (log(OR) = .479, *p* = 8.51e‐8) were significantly associated with ADHD status in the European‐ancestry matched case‐control subsample of ABCD. In the European‐ancestry subgroup of the Oregon‐ADHD‐1000 cohort, effects of negative sentiment (log(OR) = .863, *p* = 6.57e‐14) and ADHD‐PRS (log(OR) = .533, *p* = 3.56e‐8) replicated, but family income was not statistically significant (log(OR) = −.117, *p* = .186) (see Figure S6).

### Additive and interactive effects among exposures and PRS

The additive effects of all PRS and environmental exposures were examined by including all risk factors in the same regression model and applying backwards selection using a stringent cutoff (see Methods) until only those risk factors significantly and independently associated with the outcome after multiple testing remained. The effect estimates from these reduced additive‐effects models, along with *R* ^2^ values, are reported in Table 2.

**TABLE 2:** Results of the reduced additive‐effects models for total ADHD symptoms in the European‐ancestry subgroups of the ABCD and Oregon‐ADHD‐1000 cohorts.

Cohort/risk factor	Effect estimate	*p*‐value	*R* ^2^
ABCD—White/European			
ADI	0.063 (0.024, 0.102)	.001	*.028*
Family income	−0.096 (−0.129, −0.062)	2.76e‐8	*.038*
Family conflict	0.201 (0.172, 0.231)	<2e‐16	*.066*
ADHD‐PRS	0.098 (0.071, 0.125)	2.15e‐12	*.037*
Total *R* ^2^			.091
ABCD matched—White/European			
Family income	−0.125 (−0.204, −0.047)	.002	*.046*
Family conflict	0.263 (0.190, 0.337)	5.34e‐12	*.091*
ADHD‐PRS	0.195 (0.121, 0.268)	2.68e‐7	*.060*
Total *R* ^2^			.145
Oregon‐ADHD‐1000—White/European			
Negative sentiment	0.331 (0.259, 0.403)	<2e‐16	*.137*
ADHD‐PRS	0.192 (0.120, 0.263)	2.09e‐7	*.071*
Total *R* ^2^			.173

*Note*: The effect estimates (standardized regression coefficients) and *p*‐values are those from the additive model containing all risk factors listed for each cohort. *R* ^2^ values are reported for each individual risk factor (from the main effects models; *italicized*) as well as for the reduced additive model shown (Total *R* ^2^).

Abbreviations: ABCD, Adolescent Brain Cognitive Development Study; ADHD‐PRS, attention‐deficit/hyperactivity disorder‐polygenic risk score.

In the ABCD European‐ancestry subgroup, the ADHD‐PRS, ADI, family income, and family conflict were independently and additively associated with total ADHD symptoms. The additive effects of these three risk factors explained significantly more symptom variation (*R* ^2^ = .091) than any single genetic or environmental factor (max *R* ^2^ = .0663 for family conflict; all delta‐*R* ^2^ *p*‐values <2e‐16). In the Oregon‐ADHD‐1000 European‐ancestry subgroup, only the effects of ADHD‐PRS and negative sentiment replicated (*p* < .05) in the additive‐effects model (effects for ADI and income were smaller than seen in ABCD, and were not significant after adjusting for ADHD‐PRS and negative sentiment). Again, the additive model explained significantly more symptom variance (*R* ^2^ = .173) than either factor alone (max *R* ^2^ = .137 for negative sentiment; delta‐*R* ^2^ *p*‐values <2e‐7).

Comparisons of additive effects on total ADHD symptoms across the three ancestry groups in ABCD are shown in Figure S7. Differences among the ancestry groups are consistent with those seen for the univariate main effects. Meta‐analysis across the three ancestry groups showed significant additive effects for family conflict ($\hat{\beta }$ = .196, *p* < 2e‐16) and ADHD‐PRS ($\hat{\beta }$ = .084, *p* = 1.03e‐11), but effects of income (*p* = .005) and ADI (*p* = .523) were not significant (after multiple‐testing correction) due to non‐significant effects in the Hispanic/Latino and Black/African‐American groups.

Additive polygenic and environmental effects are consistent across a variety of dimensional measures of ADHD symptoms (see Tables S5–S7 and Figure S8).

For the categorical analyses (ADHD/non‐ADHD status), the additive‐effects models in the ABCD and Oregon‐ADHD‐1000 cohorts had mean AUC‐ROCs of 0.699 and 0.764, respectively (see Table S8).

**FIGURE 3:**
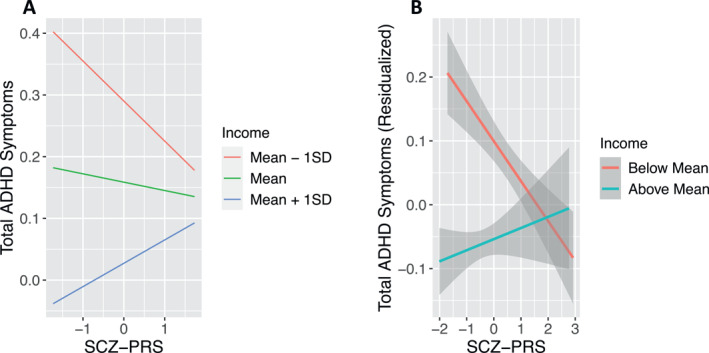
The interaction between SCZ PRS and family income is associated with total ADHD symptoms in the full ABCD cohort. Results indicate that symptoms vary depending on income to a much greater degree when genetic risk is low. (A) The marginal effects of the GxE interaction model at three values of family income: mean income and income one standard deviation above and below the mean. (B) The relationship between the residualized symptom scores (accounting for all covariates) and the SCZ‐PRS, stratified on family income (above and below the mean). ABCD, Adolescent Brain Cognitive Development Study; ADHD, attention‐deficit/hyperactivity disorder; GxE, genotype‐by‐environment interaction; SCZ‐PRS, schizophrenia‐polygenic risk score.

No interaction effects, on either dimensional or categorical outcomes, survived multiple‐testing correction in the European‐ancestry subgroup of ABCD. Nor are any interaction effects significant when meta‐analyzed across the three ancestry groups. However, in the full ABCD cohort (all participants analyzed together, regardless of ancestry) interactions between family income and PRS for SCZ, BP, CUD, and ALCDEP were significantly associated with both total ADHD and inattention symptoms. For total ADHD symptoms, the strongest interaction effect, between income and SCZ‐PRS (Figure 3), indicates that ADHD symptoms are more strongly influenced by income level when genetic risk is low. For the categorical outcome, interactions between income and both the SCZ‐PRS and CUD‐PRS, as well as an interaction between BP‐PRS and ADI, were significant. All interactions indicated that environmental effects are stronger when genetic risk is low.

However, it should be noted that interactions observed in the full ABCD cohort should be interpreted with caution, given that PRS and income/ADI are confounded with ancestry (see Figures S9 and S10). Furthermore, none of these interactions replicated in the full Oregon‐ADHD‐1000 cohort (all *p*‐values >.05).

The results of all models examining GxE interaction effects in both cohorts are reported in Tables S9–S11.

## DISCUSSION

The present study identified reproducible additive effects of genetic and environmental factors on ADHD symptoms across two large, independent cohorts. Robust effects were seen for family environment, particularly family conflict and negative sentiment, but somewhat surprisingly, not for geospatial estimated pollution exposures.

These results support the conclusion from other studies that while family processes are unlikely to be directly causal of ADHD, they do participate in maintaining and moderating symptoms over time (Harold et al., 2013; Musser et al., 2016; Peris & Hinshaw, 2003; Richards et al., 2014). Here the effect replicated well despite different methods of evaluating family process across the cohorts. While the different assessment methods (family conflict reported via the FES vs. expressed emotion derived from the FMSS) could certainly be measuring somewhat different aspects of family environment, the consistency across methods and data sets suggests a robust effect and is consistent with other research showing convergence among the FMSS expressed emotion coding and parent‐reported family conflict in regard to latent variable construct (Kim Park et al., 2008). Failure to detect main effects for pollution measures may be due to wide individual variation in actual exposure as well as metabolism and other protective factors, in these risks as they relate to ADHD. Lead exposure when measured by blood sample is well established as a risk for ADHD (Moore et al., 2022; Nigg et al., 2016) and when estimated by geolocation data was associated with neural development and cognition in ABCD (Marshall et al., 2020, 2021).

Though we identified significant PRS‐environment correlations (e.g., between ADHD‐PRS and family conflict/negative sentiment), these correlations were small (abs(*r)* <.1) and there was little attenuation of effects in additive models, indicating the PRS and environmental effects on ADHD symptoms were largely independent. However, because the ADHD‐PRS explains only a fraction of the total genetic risk for ADHD, it is likely that our findings are an underestimate of true G‐E correlation (Agnew‐Blais et al., 2022).

As expected, polygenic risk for ADHD was significantly associated with ADHD symptoms in the European‐ancestry subgroups of both cohorts (*R* ^2^ = .0365 and .0713 for ABCD and Oregon‐ADHD‐1000, respectively). New here is more evidence that additive effects of PRS and exposure measures explained markedly and significantly more symptom variance than either domain alone. This is promising and important for future efforts to develop clinical prediction algorithms. Yet, these models explained only a small fraction of the variance, confirming that additional measures will be needed beyond PRS and global exposure indices to develop such prediction algorithms for research or clinical use. Our finding that the fraction of variance explained by PRS can be increased suggests that incorporating additional measures (such as those available in the electronic medical record or population registries) may yield further increases in the accuracy of predictions.

Although we found several GxE interactions of note in the full ABCD cohort, these were not convincing due to (a) failure to replicate in the Oregon‐ADHD‐1000 cohort and (b) apparent confounding due to ancestry effects. It should be noted however that failure to see interaction effects of pollution measures with PRS scores hardly rules out the likelihood that GxE effects occur at a more granular level. For example, the effect of even low‐level lead exposure on ADHD varies depending upon genotypes in genes regulating lead metabolism (Nigg et al., 2016), suggesting that specific genes would need to be evaluated to make clinical use of GxE for some exposures. The same may prove true for air pollution measures given evidence that other pollutant exposures exert effects on neurodevelopment dependent on specific metabolizing genes (Eskenazi et al., 2014). It may also be that interaction effects for PRS do occur, but are simply too small to see at the sample sizes used here.

The observed differences among ancestry groups highlight the need for study of risk factors, both environmental and genetic, in diverse samples. The lack of PRS associations in the Black/African‐American subgroup is consistent with previous evidence that polygenic scores derived from European populations generally perform poorly among populations with African ancestry (Duncan et al., 2019), and reiterates the need for large‐scale GWAS in non‐European ancestry populations to improve the generalizability of PRS and avoid health care disparities should PRS eventually become useful in clinical prediction algorithms.

Our use of multiple PRS, geocoded environmental exposures, and two large cohorts to assess reproducibility of effects are important contributions to the literature on gene‐environment interplay related to ADHD. However, several limitations of the current study should be considered. First, we focused on exposures during childhood (concurrent with symptom measures). It is likely that toxicants play a role much earlier in development (Block et al., 2012; Han et al., 2021; Thygesen et al., 2020), which could explain the lack of main effect associations and GxE effects seen here. Second, given the cross‐sectional design of our study, we are unable to comment on the causal direction of effects. For example, bidirectional effects between ADHD symptoms and family conflict are likely.

Future studies on the interplay between genetic and environmental risk factors for ADHD should consider longitudinal measures of exposures from the perinatal period through childhood, as effects likely vary across development, and should pay special attention to differences in risk factors dependent on race/ethnicity. Future work should also determine if the use of machine learning models, including more advanced model selection techniques, could improve the accuracy of the predictions reported here.

In conclusion, the present study adds to the literature finding that interactions between PRS and particular exposures are elusive and likely small for mental disorders in children. However, other types of interactions that are theoretically driven remain in need of examination. At the same time, results confirm association between ADHD‐PRS and ADHD (*R* ^2^ ∼.04–.07), and association between family conflict/negative sentiment and ADHD independent of polygenic risk for psychopathology. Finally, it supports the utility of combining environmental exposure measures and PRS in future prediction algorithms.

## AUTHOR CONTRIBUTIONS

**Megan M. Herting**, **Stephen V. Faraone**, **Michael A. Mooney**, **Joel T. Nigg**: Conceptualization. **Megan M. Herting**, **Michael A. Mooney**, **Joel T. Nigg**, **Peter Ryabinin**, **Joel Schwartz**, **Katharine Selah**: Methodology. **Dylan Antovich**, **Michael Kozlowski**, **Michael A. Mooney**, **Hannah Morton**, **Peter Ryabinin**, **Katharine Selah**: Formal Analysis. **Megan M. Herting**, **Joel T. Nigg**, **Joel Schwartz**: Resources. **Hannah Morton**, **Elizabeth Nousen**, **Jessica Tipsord**: Data Curation. **Michael A. Mooney**, **Peter Ryabinin**: Visualization. **Joel T. Nigg**, **Jessica Tipsord**: Project Administration. **Michael A. Mooney**, **Joel T. Nigg**: Supervision. **Joel T. Nigg**: Funding Acquisition. **Michael A. Mooney**, **Joel T. Nigg**, **Peter Ryabinin**: Writing – Original Draft. All authors: Writing – Review & Editing.

## CONFLICT OF INTEREST STATEMENT

Stephen V. Faraone and Joel T. Nigg both serve on the JCPP Advances Editorial Advisory Board. The remaining authors have declared they have no competing or potential conflicts of interest.

## ETHICAL CONSIDERATIONS

Human subjects and ethics approval was obtained from the local University Institutional Review Board. A parent/legal guardian provided written informed consent, and children provided informed written assent.

## Supporting information

Supporting Information S1

## Data Availability

The data that support the findings of this study are openly available in the NIMH Data Archive at https://nda.nih.gov/study.html?id=1299 and https://nda.nih.gov/edit_collection.html?id=2857.
