# Supplementary material for: Joint polygenic and environmental risks for childhood attention‐deficit/hyperactivity disorder (ADHD) and ADHD symptom dimensions
Source: JCPP Adv. 2023 Mar 16;3(2):e12152. doi: 10.1002/jcv2.12152 (PMC10519744; doi:10.1002/jcv2.12152)
Supplement: Supplementary file 1 — Supporting Information S1 [file JCV2-3-e12152-s001.docx]

SUPPORTING INFORMATION

Joint Polygenic and Environmental Risks for Childhood Attention-Deficit/Hyperactivity Disorder (ADHD) and ADHD Symptom Dimensions

Michael A. Mooney^1,2^*, Peter Ryabinin^2^*, Hannah Morton^3^, Katharine Selah^3^, Rose Gonoud^3^, Michael Kozlowski^3^, Elizabeth Nousen^3^, Jessica Tipsord^3^, Dylan Antovich^3^, Joel Schwartz^4^, Megan M. Herting^5,6^, Stephen V. Faraone^7^, Joel T. Nigg^3,8^

1. Division of Bioinformatics and Computational Biology, Department of Medical Informatics and Clinical Epidemiology, Oregon Health & Science University, Portland, Oregon, USA

2. Knight Cancer Institute, Oregon Health & Science University, Portland, Oregon, USA

3. Center for ADHD Research, Department of Psychiatry, Oregon Health & Science University, Portland, Oregon, USA

4. Department of Environmental Health, Harvard T.H. Chan School of Public Health, Boston, MA, USA.

5. Department of Population and Public Health Sciences, Keck School of Medicine of the University of Southern California, Los Angeles, California, USA

6. Department of Pediatrics, Children’s Hospital Los Angeles, Los Angeles, California, USA

7. Department of Psychiatry, SUNY Upstate Medical University, Syracuse, New York, USA

8. Department of Behavioral Neuroscience, Oregon Health & Science University, Portland, Oregon, USA

*Contributed equally to this work.

**Methods:**

*ADHD Assessment:*

In the Oregon-ADHD-1000, ADHD diagnosis was performed using a multi-method, multi-informant, multi-reviewer best-estimate protocol that included community outreach and recruitment (to avoid clinical setting referral bias), standardized, nationally-normed rating scales from parent and teacher, parent semi-structured clinical interview administered by a clinically trained interviewer with adequate inter-interviewer reliability, child intellectual testing, and clinical observation and notes from two trained research assistants. Two clinicians (board certified child psychiatrist and a licensed child clinical psychologist) reviewed all available data independently to arrive at an ADHD/non-ADHD decision, with adequate agreement (k>0.80). Disagreements were resolved by consensus. The full diagnostic assessment procedure has been described previously (Nigg et al., 2018).

In the ABCD cohort, ADHD/non-ADHD status was based on the Tier 4 criteria described by Cordova *et al.* (Cordova et al., 2022), but was expanded to include participants who met diagnostic criteria in the past and still had elevated symptoms. Specifically, ADHD cases were those who met criteria on the computerized Kiddie Schedule for Affective Disorders and Schizophrenia for School-Age Children (KSADS) interview as well as exceeding cutoffs on parent and teacher normative ratings. In addition, participants who met diagnostic criteria for ADHD in the past (computerized KSADS), but despite not meeting criteria on the interview currently remained on ADHD medication, and had an elevated attention problems T-score from the teacher-reported Brief Problems Monitor (T-score > 65), suggesting that they were in fact true cases who were partially treated, causing them not to meet full criteria. Cordova *et al.* describe this in their supplemental materials and note that adding these cases provides a viable alternative without compromising genetic validity/correlations (Cordova et al., 2022).

*Group Matching in ABCD:*

A matched case-control subsample of the ABCD cohort was defined as follows. Participants identified as ADHD cases (N=540) were matched to two randomly selected non-ADHD study participants using the MatchIt R package (Ho et al., 2011). Children were matched exactly on sex, race, ethnicity and study site, and were “anti-matched” on family (meaning cases and matched controls were not allowed to be from the same family). A small number of ADHD cases who did not match a non-ADHD subject using the above criteria, were matched without regard to race (N=17), or ethnicity (N=1). Age was matched to the closest value possible, while ensuring the mean age of the ADHD and non-ADHD groups were approximately equal. For ancestry-stratified analyses, these matches were filtered to include only European-ancestry participants, resulting in 240 ADHD cases and 492 controls.

*Polygenic Risk Scores:*

The sources of the GWAS summary statistics used for constructing all polygenic risk scores examined in the study are provided in Table S1. All results were accessed from the Psychiatric Genomics Consortium website (<https://www.med.unc.edu/pgc/download-results/>).

*Family Environment:*

Family income was encoded as an ordinal variable using the following categories in the ABCD cohort: <$5,000, $5,000-$11,999, $12,000-$15,999, $16,000-$24,999, $25,000-$34,999, $35,000-$49,999, $50,000-$74,999, $75,000-$99,999, $100,000-$199,999, and ≥$200,000. In the Oregon-ADHD-1000 cohort, family income was encoded as: <$25,000, $25,000-$34,999, $35,000-$49,999, $50,000-$74,999, $75,000-$99,999, $100,000-$129,999, $130,000-$149,999, and ≥$150,000.

In the Oregon-ADHD-1000 cohort, parental expressed emotion and implicit sentiment were assessed via the Five Minute Speech Sample (FMSS) (Magaña et al., 1986). Following standard procedures, parents were asked to describe their child and their relationship with their child for five minutes, in their own words, without interruption, while being audio recorded. Transcripts of these recordings were then used to generate measures of negative emotional tone, based on pre-trained computerized text classification models from the open-source Transformers library (HuggingFace, 2016). Specifically, three Bidirectional Encoder Representations, also known as BERTs (Devlin et al., 2019), were utilized. For the purposes of modeling sentiment, these models were trained on data from film review websites, such as IMDb or Rotten Tomatoes, or the Stanford Sentiment Treebank (SST-2) (Socher et al., 2013), a data set created specifically to test models used for sentiment analysis. To obtain a single measure of sentiment/emotional tone for each FMSS transcript, each language model was applied to each sentence in the transcript, producing a probability that the sentence contains negative sentiment. The probability of negative sentiment in each sentence was averaged across all sentences in the transcript to obtain a mean negative sentiment score. The final measure of negative sentiment/emotional tone was based on a standardized composite of scores from three models, two trained on the Rotten Tomatoes data, and one trained on the Stanford Sentiment Treebank, with a higher score indicating more negative sentiment (Selah et al., under review).

*Examination of Ancestry-specific Effects:*

Genomic principal components (PCs) were calculated by combining each cohort (separately) with 1301 HapMap samples and iteratively applying PC-AiR and PC-Relate until genetic relatedness (3^rd^ degree or closer) of all samples was unchanged between two successive iterations, or a maximum of 20 iterations was reached. The PCs from the final iteration of PC-AiR were used as the genomic PCs in the primary analyses reported. PCs 1 and 2 were used to define a homogeneous European-ancestry subgroup. This was done by identifying the centroid, in the plane of PCs 1 and 2, of those subjects who self-identified as White and Non-Hispanic. The root mean squared error (RMSE) relative to this centroid was calculated across all subjects, and only those within 1 RMSE of the centroid were assigned to the homogeneous European-ancestry subgroup. The other ancestry groups were defined as those that did not meet the above criteria (i.e., non-European) and self-identified as either Hispanic/Latino or Black/African-American. Figure S1 shows the genomic principal components for all three ancestry subgroups in ABCD. All subgroups align well with corresponding ancestry populations from HapMap. PCs 1-10 were included as covariates in regression models.

See below for a sensitivity analysis examining the effect of re-calculating PCs within each ancestry subgroup.

*Missing Data Imputation:*

The amount of missing data present among the variables analyzed is shown in Table S2 (variables not shown had complete data). Imputation of missing data for all variables was performed using multiple imputation as implemented in the *mice* R package. Predictive mean matching (PMM) was used for the imputation and 50 imputation sets were created. In addition to the primary variables for the models, the auxiliary variables included to support the imputation model are listed in Table S3.

See below for results of a sensitivity analysis examining the effect of imputing the PRS.

*Sensitivity Analyses*:

*Variable Scaling:*

All PRS, exposure, and quantitative ADHD variables were normalized to a Z-score (mean 0, standard deviation 1) prior to modeling. A sensitive analysis was performed to determine if instead normalizing measures using a rank-based inverse normal transformation, implemented using the RNOmni R package (McCaw et al., 2020), meaningfully impacted the observed associations.

No appreciable differences were seen between the two data transformation methods. For instance, for the univariate main effects models on total ADHD symptoms in the European-ancestry subgroup of ABCD, effect estimates produced with the two methods were nearly identical (*r*=0.997), and there was only one difference in terms of statistical significance of individual effects (ALCDEP-PRS p=0.00485 vs. 0.000194). No differences were seen in terms of additive effects.

*Relatedness Among Participants:*

Family sizes based on self-reported sibling relationships are shown in Table S4. There is evidence of more distant relatedness (e.g., 3^rd^ degree relations) as well as a small amount of cryptic relatedness, in both cohorts, based on the genetic data. Distant and cryptic relatedness would not be accounted for by the GEE models used in our analyses, which relied on the reported family IDs to cluster participants. To examine the effects of the small amount of distant and cryptic relatedness, analyses were repeated within an unrelated sub-sample identified based on genotype data. This sub-sample was created by retaining only one participant from groups determined to be genetically related (3^rd^ degree or closer).

Results from analyses in this unrelated sub-sample confirm the primary results reported. For instance, the reduced additive-effects model in the ABCD European-ancestry, unrelated sub-sample (N=4467) confirms that the ADHD-PRS ($\hat{\beta}$=0.103), family conflict ($\hat{\beta}$=0.199), family income ($\hat{\beta}$=-0.094), and ADI ($\hat{\beta}$=0.080) are all significantly associated with total ADHD symptoms, with effect estimates not meaningfully different from those of the primary analyses (all estimates within the 95% confidence intervals of the primary estimates).

*Imputation of PRS:*

To determine whether imputing missing PRS values significantly impacted GxE interaction effects observed in the full ABCD cohort, models were recomputed using only samples with complete PRS data. Significant interaction effects between family income and PRS for SCZ, BP, CUD and ALCDEP were confirmed: Income x SCZ-PRS ($\hat{\beta}$=0.050, p=5.22e-5), Income x BP-PRS ($\hat{\beta}$=0.050, p=4.17e-5), Income x CUD-PRS ($\hat{\beta}$=0.048, p=3.23e-5) and Income x ALCDEP-PRS ($\hat{\beta}$=0.043, p=4.60e-4). Note: ancestry subgroups were selected from samples with genetic data, so imputation of missing PRS was done only for analyses of the full cohorts (all participants, regardless of ancestry).

*Genomic Principal Components:*

When conducting ancestry-stratified analyses, recalculating genomic PCs within each ancestry group may provide additional correction for fine population structure or technical artifacts (e.g., batch effects). Therefore, for the ancestry-specific analyses reported here, we conducted additional analyses to determine whether covarying genomic PCs computed across all samples (“global” PCs) vs. PCs computed within each individual ancestry subgroup affected our findings. Results of models that included re-calculated (ancestry-specific) PCs did not show any appreciable differences from the primary results reported. For instance, results for the reduced additive-effects model (total ADHD symptoms as outcome) in the ABCD European-ancestry were nearly identical when the re-calculated PCs were included as covariates: ADHD-PRS ($\hat{\beta}$=0.100), family conflict ($\hat{\beta}$=0.201), family income ($\hat{\beta}$=-0.094), and ADI ($\hat{\beta}$=0.062).

*Assessing Consistency of Results Across Multiple Symptom Measures:*

We conducted additional analyses using a variety of measures of ADHD symptoms to examine the consistency of genetic and environmental effects across outcomes. In the ABCD cohort, we examined inattention and hyperactivity symptoms from the computerized Kiddie Schedule of Affective Disorders and Schizophrenia (KSADS-COMP). In the Oregon cohort, we examined Conners 3^rd^ edition inattention and hyperactivity T-scores, parent-reported ADHD Rating Scale inattention and hyperactivity T-scores, as well as KSADS inattention and hyperactivity symptom counts. Genetic and environmental effects were consistent across all measures, except that effect sizes for all risk factors were smaller for the KSADS-COMP measures in ABCD. Results for the reduced additive effects models in the European-ancestry subgroups of each cohort are shown in Tables S6 and S7.

**Tables / Figures:**

| Disorder | Sample  Size | Label / Data Set Name | File Name /  Paper Link |
| --- | --- | --- | --- |
| ADHD | 55374 | ADHD /  adhd2019 | daner_adhd_meta_filtered_NA_iPSYCH23_PGC11_sigPCs_woSEX_2ell6sd_Neff_70.meta.gz /  <https://pubmed.ncbi.nlm.nih.gov/30478444/> |
| Anxiety | 17310 | ANX /  anx2016 | anxiety.meta.full.cc.tbl.gzip /  <https://pubmed.ncbi.nlm.nih.gov/26754954/> |
| Autism | 46350 | ASD /  asd2019 | iPSYCH-PGC_ASD_Nov2017.gz /  <https://pubmed.ncbi.nlm.nih.gov/30804558/> |
| Bipolar | 413466 | BIP /  bip2021 | pgc-bip2021-all.vcf.tsv.gz <https://pubmed.ncbi.nlm.nih.gov/34002096/> |
| MDD | 807553 | MDD /  mdd2019endinburgh | PGC_UKB_depression_genome-wide.txt /  <https://pubmed.ncbi.nlm.nih.gov/30718901/> |
| Schizophrenia | 105318 | SCZ /  scz2018clozuk | CLOZUK_PGC2noclo.METAL.assoc.dosage.fix.gz /  <https://pubmed.ncbi.nlm.nih.gov/29483656/> |
| Alcohol Dependence | 52848 | ALCDEP /  sud2018-alc | pgc_alcdep.trans_fe_unrel_geno.aug2018_release.txt.gz /  <https://pubmed.ncbi.nlm.nih.gov/30482948/> |
| Alcohol Use Disorder | 121604 | AUD /  sud2019-alcuse | AUDIT_UKB_2018_AJP.txt.gz /  <https://pubmed.ncbi.nlm.nih.gov/30336701/> |
| Cannabis Use Disorder | 374287 | CUD /  sud2020-cud | CUD_EUR_casecontrol_public_11.14.2020.gz / <https://pubmed.ncbi.nlm.nih.gov/33096046/> |

Table S1. GWAS discovery data sets for the polygenic risk scores used in the current study. All results were downloaded from: <https://www.med.unc.edu/pgc/download-results/>

| Cohort | Inattention Symptoms | Total ADHD Symptoms | Genotype | ADI | Family Income | Family Conflict | Lead Risk | NO2 | PM2.5 | Age |
| --- | --- | --- | --- | --- | --- | --- | --- | --- | --- | --- |
| ABCD - All | 8 (0.0674%) | 8 (0.0674%) | 1370 (11.5%) | 928 (7.82%) | 1017 (8.57%) | 4 (0.0337%) | 703 (5.92%) | 647 (5.45%) | 647 (5.45%) | 0 (0%) |
| ABCD - White/European | 3 (0.0539%) | 3 (0.0539%) | 0 (0%) | 373 (6.71%) | 254 (4.57%) | 0 (0%) | 255 (4.58%) | 222 (3.99%) | 222 (3.99%) | 0 (0%) |
| ABCD - Black/African-American | 0 (0%) | 0 (0%) | 0 (0%) | 162 (11.4%) | 204 (14.3%) | 0 (0%) | 122 (8.57%) | 116 (8.15%) | 116 (8.15%) | 0 (0%) |
| ABCD - Hispanic/Latino | 2 (0.18%) | 2 (0.18%) | 0 (0%) | 71 (6.4%) | 131 (11.8%) | 1 (0.0901%) | 53 (4.77%) | 52 (4.68%) | 52 (4.68%) | 0 (0%) |
| Oregon - All | 23 (1.59%) | 23 (1.59%) | 679 (46.9%) | 5 (0.345%) | 102 (7.04%) | 617 (42.6%) | 3 (0.207%) | 38 (2.62%) | 2 (0.138%) | 31 (2.14%) |
| Oregon - White/European | 3 (0.493%) | 4 (0.657%) | 0 (0%) | 3 (0.493%) | 36 (5.91%) | 35 (5.75%) | 2 (0.328%) | 15 (2.46%) | 2 (0.328%) | 6 (0.985%) |

Table S2. Number of missing data values for each cohort and subsample. Variables with no missing data are not shown.

| Oregon-ADHD-1000 | | ABCD | |
| --- | --- | --- | --- |
| Variable | Description | Variable | Description |
| sex_parent | Sex of primary parent | demo_ethn_v2 | Do you consider the child Hispanic/Latino/Latina? |
| childrace | Child race | demo_gender_id_v2 | What is the child's current gender identity? |
| child_ethnicity | Child ethnicity | demo_prnt_ed_v2 | What is the highest grade or level of school you have completed or the highest degree you have received? |
| y1_p_edu | Parent highest level of education | demo_prnt_empl_v2 | Are you working now, looking for work, retired, stay at home parent, a student, or something else? |
| y1_highest_rel_job_score | Highest Job Score in Adults in child's household: scored according to  Nam-Powers occupational status scores from the 2000 census (1 is low status, 100 is highest status) | demo_prnt_gender_id_v2 | What is your current gender identity? |
| y1_marital_status | Marital status of child’s biological parents | demo_prnt_income_v2 | How much did you earn, before taxes and other deductions, during the past 12 months? |
| y1_cu_adhdmed | Current ADHD medication (stimulant or non-stimulant) use (Yes/No) | demo_prnt_marital_v2 | Are you now married, widowed, divorced, separated, never married or living with a partner? |
| y1_t_adhdrs_tot_ts | Teacher-reported child ADHDRS total symptom T-score | demo_race_a_p___10 | What race do you consider the child to be? (10 White Blanca) |
| y1_p1_s_cu_adhdrs_hypsx | Primary parent self-reported current ADHDRS hyperactivity symptoms | demo_race_a_p___11 | What race do you consider the child to be? (11, Black/African American Negra o afroamericana) |
| y1_p1_s_cu_adhdrs_intsx | Primary parent self-reported current ADHDRS inattention symptoms | combined_race_dcan | Factor inclusive of all race options in ABCD |
| y1_p_p1_adhdrs_intsx | Parent reporting on primary parent ADHDRS inattention symptoms | predictedethnicityrmse1 | European-ancestry (Yes/No) derived from genetic data |
| y1_p_p1_adhdrs_hypsx | Parent reporting on primary parent ADHDRS hyperactivity symptoms | demo_roster_v2 | How many people are living at your address? INCLUDE everyone who is living or staying at your address for more than 2 months. |
| y1_ksad_intsx | Child KSADS inattention symptoms | acs_raked_propensity_score | Raked propensity score for weighting (Heeringa & Berglund, 2020) |
| y1_ksad_hypsx | Child KSADS hyperactivity symptoms | adhd_medicationuse | ADHD medication use |
| y1_ksad_intsx_sub | Child KSADS inattention symptoms, including subthreshold | asr_scr_adhd_t | Parent Adult Self Report Scores Aseba  AD/H Problems ASR DSM-5-Oriented Scale (t-score) |
| y1_ksad_hypsx_sub | Child KSADS hyperactivity symptoms, including subthreshold | asr_scr_attention_t | Attention Problems ASR Syndrome Scale (t-score) |
| y1_c_lt_ksad_adhdimp | Child KSADS lifetime ADHD impairment | asr_scr_hyperactive_t | Hyperactivity-Impulsivity ASR DSM-5-Oriented Scale (t-score) |
| y1_c_lt_ksad_oddimp | Child KSADS lifetime ODD impairment | asr_scr_inattention_t | Inattention ASR DSM-5-Oriented Scale (t-score) |
| y1_ksad_oddsx_sub | Child KSADS ODD symptoms, including subthreshold | bpmt_scr_attention_t | Teacher-reported child BPM attention T-score |
| y1_c_cu_ksad_oddimp | Child KSADS current ODD impairment | cbcl_scr_syn_external_t | CBCL external syndrome T-score |
| respnum_ksad | KSAD Parent Respondent Identifier | cbcl_scr_syn_internal_t | CBCL internal syndrome T-score |
| y1_p_con3_ag_ts | Parent-reported child Conners 3 aggression T-score | ksad_asdsum | # of ASD symptoms endorsed on KSADS |
| y1_p_con3_ef_ts | Parent-reported child Conners 3 executive function T-score | ksads_1_840_p | Diagnosis - Major Depressive Disorder Present - Parent report |
| y1_p_con3_lp_ts | Parent-reported child Conners 3 learning problems T-score | ksads_1_840_t | Diagnosis - Major Depressive Disorder Present - Youth report |
| y1_p_con3_pr_ts | Parent-reported child Conners 3 peer relations T-score | ksads_1_841_p | Diagnosis - Major Depressive Disorder Current in Partial Remission (F32.4) - Parent report |
| y1_t_c_con3_int_ts | Teacher-reported child Conners 3 inattention T-score | ksads_1_841_t | Diagnosis - Major Depressive Disorder Current in Partial Remission (F32.4) - Youth report |
| y1_t_c_con3_hyp_ts | Teacher-reported child Conners 3 inattention T-score | ksads_1_842_p | Diagnosis - Major Depressive Disorder Past (F32.9) - Parent report |
| y1_t_c_con3_le_ts | Teacher-reported child Conners 3 learning problems T-score | ksads_1_842_t | Diagnosis - Major Depressive Disorder Past (F32.9) - Youth report |
| y1_t_c_con3_ag_ts | Teacher-reported child Conners 3 aggression T-score | ksads_1_843_p | Diagnosis - Persistent Depressive Disorder (Dysthymia) Present F34.1 - Parent report |
| y1_t_c_con3_pr_ts | Teacher-reported child Conners 3 peer relations T-score | ksads_1_843_t | Diagnosis - Persistent Depressive Disorder (Dysthymia) Present F34.1 - Youth report |
| y1_t_c_con3_pi_vs | Teacher-reported child Conners 3 positive impression validity scale | ksads_1_844_p | Diagnosis - Persistent Depressive Disorder (Dysthymia) In Partial Remission F34.1 - Parent report |
| y1_t_c_con3_ni_vs | Teacher-reported child Conners 3 negative impression validity scale | ksads_1_844_t | Diagnosis - Persistent Depressive Disorder (Dysthymia) In Partial Remission F34.1 - Youth report |
| y1_tmcq_activcont | TMCQ activation control scale | ksads_1_845_p | Diagnosis - Persistent Depressive Disorder (Dysthymia) Past F34.1 - Parent report |
| y1_tmcq_activity | TMCQ activity level scale | ksads_1_845_t | Diagnosis - Persistent Depressive Disorder (Dysthymia) Past F34.1 - Youth report |
| y1_tmcq_affil | TMCQ affiliation scale | ksads_1_846_p | Diagnosis - Unspecified Depressive Disorder Current (F32.9) - Parent report |
| y1_tmcq_anger | TMCQ anger / frustration scale | ksads_1_846_t | Diagnosis - Unspecified Depressive Disorder Current (F32.9) - Youth report |
| y1_tmcq_fear | TMCQ fear scale | ksads_1_847_p | Diagnosis - Unspecified Depressive Disorder Past (F32.9) - Parent report |
| y1_tmcq_hip | TMCQ high intensity pleasure scale | ksads_1_847_t | Diagnosis - Unspecified Depressive Disorder Past (F32.9) - Youth report |
| y1_tmcq_impuls | TMCQ impulsivity scale | ksads_10_869_p | Diagnosis - Generalized Anxiety Disorder Present (F41.1) - Parent report |
| y1_tmcq_inhibit | TMCQ inhibitory control scale | ksads_10_869_t | Diagnosis - Generalized Anxiety Disorder Present (F41.1) - Youth report |
| y1_tmcq_sad | TMCQ sadness scale | ksads_10_913_p | Diagnosis - Other Specified Anxiety Disorder (Generalized Anxiety Disorder impairment does not meet minimum duration) F41.8 - Parent report |
| y1_tmcq_shy | TMCQ shyness scale | ksads_10_913_t | Diagnosis - Other Specified Anxiety Disorder (Generalized Anxiety Disorder impairment does not meet minimum duration) F41.8 - Youth report |
| y1_tmcq_soothe | TMCQ soothability / falling reactivity scale | ksads_11_917_p | Diagnosis - Obsessive-Compulsive Disorder Present (F42) - Parent report |
| y1_tmcq_assert | TMCQ assertiveness / dominance scale | ksads_14_81_p | Symptom - Easily distracted since elementary school Present |
| y1_tmcq_attfocus | TMCQ attention focusing scale | ksads_14_84_p | Symptom - Difficulty remaining seated Present |
| y1_tmcq_lip | TMCQ low intensity pleasure scale | ksads_14_853_p | Diagnosis - Attention-Deficit/Hyperactivity Disorder Present - Parent report |
| y1_tmcq_percept | TMCQ perceptual sensitivity scale | ksads_14_854_p | Diagnosis - Attention-Deficit/Hyperactivity Disorder Past |
| y1_tmcq_discomf | TMCQ discomfort scale | ksads_14_855_p | Diagnosis - Attention-Deficit/Hyperactivity Disorder IN PARTIAL REMISSION |
| y1_tmcq_openness | TMCQ fantasy / openness scale | ksads_15_901_p | Diagnosis - Oppositional Defiant Disorder Present F91.3 - Parent report |
| y1_tmcq_surgency | TMCQ surgency scale | ksads_15_902_p | Diagnosis - Oppositional Defiant Disorder Past F91.3 - Parent report |
| y1_tmcq_effcont | TMCQ effortful control scale | ksads_16_897_p | Diagnosis - Conduct Disorder present childhood onset (F91.1) - Parent report |
| y1_tmcq_negaffect | TMCQ negative affect scale | ksads_16_898_p | Diagnosis - Conduct Disorder present adolescent onset (F91.2) - Parent report |
| shyr | TMCQ shyness scale – reversed | ksads_2_830_p | Diagnosis - Bipolar I Disorder current episode manic (F31.1x) - Parent report |
| soother | TMCQ soothability scale – reversed | ksads_2_830_t | Diagnosis - Bipolar I Disorder current episode manic (F31.1x) - Youth report |
| y1_p_sdq_co_ts | Parent SDQ conduct problems T-score | ksads_2_833_p | Diagnosis - Bipolar I Disorder most recent past episode manic (F31.1x) - Parent report |
| y1_p_sdq_px_ts | Parent SDQ total problems T-score | ksads_2_833_t | Diagnosis - Bipolar I Disorder most recent past episode manic (F31.1x) - Youth report |
| y1_t_c_sdq_em_ts | Teacher SDQ emotional problems T-score | ksads_21_921_p | Diagnosis - Post-Traumatic Stress Disorder Present (F94.1) - Parent report |
| y1_t_c_sdq_co_ts | Teacher SDQ conduct problems T-score | ksads_21_923_p | Diagnosis - Other Specified Trauma-and Stressor-Related Disorder present (PTSD impairment does not meet full criteria (F43.8) - Parent report |
| y1_t_c_sdq_hy_ts | Teacher SDQ hyperactivity / inattention T-score | ksads_3_848_p | Diagnosis - Disruptive Mood Dysregulation Disorder (DMDD) Current (F34.8) - Parent report |
| y1_t_c_sdq_pe_ts | Teacher SDQ peer problems T-score | ksads_3_848_t | Diagnosis - Disruptive Mood Dysregulation Disorder (DMDD) Current (F34.8) - Youth report |
| y1_t_c_sdq_so_ts | Teacher SDQ prosocial behavior T-score | ksads_4_826_p | Diagnosis - Hallucinations (Present) - Parent report |
| y1_t_c_sdq_px_ts | Teacher SDQ total problems T-score | ksads_4_828_p | Diagnosis - Delusions (Present) - Parent report |
| y1_t_c_sdq_im_ts | Teacher SDQ impact of problems T-score | ksads_4_849_p | Diagnosis - Associated Psychotic Symptoms Current - Parent report |
| y1_p1_s_scid_adhd | Primary parent ADHD Diagnosis based on SCID | ksads_4_851_p | Diagnosis - Unspecified Schizophrenia Spectrum and Other Psychotic Disorder F29 (Current) - Parent report |
| y1_p1_s_lt_scid_intsx_sub | Primary Parent on Self SCID: ADHD lifetime # of inattentive symptoms (subthreshold counted) | ksads_5_857_p | Diagnosis - Panic Disorder (F41.0) Present - Parent report |
| y1_p1_s_lt_scid_hypsx_sub | Primary Parent on Self SCID: ADHD lifetime # of hyperactive symptoms (subthreshold counted) | ksads_5_906_p | Diagnosis - Other Specified Anxiety Disorder (Panic Disorder impairment does not meet full criteria) F41.8 - Parent report |
| y1_p1_s_cu_scid_intsx_sub | Primary Parent on Self SCID: ADHD current # of inattentive symptoms (subthreshold counted) | ksads_6_859_p | Diagnosis - Agoraphobia (F40.00) Present - Parent report |
| y1_p1_s_cu_scid_hypsx_sub | Primary Parent on Self SCID: ADHD current # of hyperactive symptoms (subthreshold counted) | ksads_6_908_p | Diagnosis - Other Specified Anxiety Disorder (Agoraphobia impairment does not meet full criteria) F41.8 - Parent report |
| y1_cdi_tot_ts | Child depression: CDI total T-score | ksads_7_861_p | Diagnosis - Separation Anxiety Disorder (F93.00) Present - Parent report |
| y1_scq_tot_score | Child autism symptoms total score SCQ (sum of items 2-40) | ksads_7_909_p | Diagnosis - Other Specified Anxiety Disorder (Separation Anxiety Disorder impairment does not meet full criteria) F41.8 - Parent report |
| y1_p1_asr_att_ts | ASR Primary Parent attention problems T-score | ksads_8_863_p | Diagnosis - Social Anxiety Disorder (F40.10) Present - Parent report |
| y1_p1_s_caars_a_ts | CAARS inattention / memory problems T-score, Primary parent reporting on self | ksads_8_863_t | Diagnosis - Social Anxiety Disorder (F40.10) Present - Youth report |
| y1_p1_s_caars_b_ts | CAARS hyperactivity / restlessness problems T-score, Primary parent reporting on self | ksads_8_911_p | Diagnosis - Other Specified Anxiety Disorder (Social Anxiety Disorder impairment does not meet minimum duration) F41.8 - Parent report |
| y1_p1_s_caars_h_ts | Primary Parent on Self CAARS DSM-IV ADHD symptoms T-score | ksads_8_911_t | Diagnosis - Other Specified Anxiety Disorder (Social Anxiety Disorder impairment does not meet minimum duration) F41.8 - Youth report |
| y1_p_p1_parenta_ts | Other parent rating of primary parent CAARS Inattention / Memory Problems T-score | ksads_9_867_p | Diagnosis - Specific Phobia Present (F40.2XX) - Parent report |
| y1_p_p1_parentb_ts | Other parent rating of primary parent CAARS Hyperactivity / Restlessness T-score | lmt_scr_perc_correct | Little Man Test percentage correct of all 32 presented trials |
| y1_p_p1_parente_ts | Other parent rating of primary parent CAARS ADHD Index T-score | nihtb_pc1 | General ability principal component (Thompson et al., 2019) |
| y1_masc_socanx_ts | MASC social anxiety T-score | nihtb_pc2 | Executive function principal component (Thompson et al., 2019) |
| y1_masc_adi_ts | MASC ADI T-score | nihtb_pc3 | Learning / Memory principal component (Thompson et al., 2019) |
| y1_wisc_voc_ss | Vocabulary standard score WISC-IV on child | nihtbx_cardsort_uncorrected | NIH Toolbox Dimensional Change Card Sort Test Ages 8-11 v2.0 Uncorrected Standard Score |
| y1_wisc_fsiq | Full Scale IQ on child for 3 subtests --information, vocabulary, block design WISC-IV | nihtbx_flanker_uncorrected | NIH Toolbox Flanker Inhibitory Control and Attention Test Ages 8-11 v2.0 Uncorrected Standard Score |
| y1_wiat_read_ss | Reading standard score WIAT-II on child | nihtbx_list_uncorrected | NIH Toolbox List Sorting Working Memory Test Age 7+ v2.0 Uncorrected Standard Score |
| y1_p_f_fes_conf_ss | FES conflict subscale | nihtbx_pattern_uncorrected | NIH Toolbox Pattern Comparison Processing Speed Test Age 7+ v2.0 Uncorrected Standard Score |
| drift_rate_y1 | Stop-Go Task Drift Rate | nihtbx_picture_uncorrected | NIH Toolbox Picture Sequence Memory Test Age 8+ Form A v2.0 Uncorrected Standard Score |
| stop_ssrtave_y1 | Stop-Go Task SSRT | nihtbx_picvocab_uncorrected | NIH Toolbox Picture Vocabulary Test Age 3+ v2.0 Uncorrected Standard Score |
| dprime1_y1 | Signal detection parameter d-prime, catch trials | nihtbx_reading_uncorrected | NIH Toolbox Oral Reading Recognition Test Age 3+ v2.0 Uncorrected Standard Score |
| dprime2_y1 | Signal detection parameter d-prime, standard trials | ravlt_sumtotal | Pearson RAVLT sum of Short Delay trials 1-5, total correct |
| ssbk_numcomplete_y1 | Spatial Span Backward | oddsx_total | Composite parent KSADS ODD symptoms (Cordova et al., 2022) |
| ssfd_numcomplete_y1 | Spatial Span Forward | p_adhd | Composite parent ADHD score (Cordova et al., 2022) |
| n1backacc_y1 | N-Back Task (1-back) | p_irrit | Composite parent irritability score (Cordova et al., 2022) |
| n2backacc_y1 | N-Back Task (2-back) | p_ksad_hyp | Composite parent KSADS hyperactivity score (Cordova et al., 2022) |
| y1_digits_bkwd_ss | Digit Span Backward | p_ksad_int | Composite parent KSADS inattention score (Cordova et al., 2022) |
| y1_digits_frwd_ss | Digit Span Forward | pea_wiscv_tss | WISC-V matrix reasoning total scaled score |
| y1_clwrd_cond1 | Stroop Task – Color | srs_sum | Sum of Social Responsiveness Scale short form |
| y1_clwrd_cond2 | Stroop Task – Word | t_adhd | Composite teacher ADHD (Cordova et al., 2022) |
| y1_clwrd_res | Stroop color word interference trial with residual score regressed on Stroop color and word naming (Nigg et al., 2018) | t_dbd | Composite teacher ODD/CD score (Cordova et al., 2022) |
| y1_trails_cond2 | Trail Making Task – Condition 2 | t_mood | Composite teacher internalizing/mood score (Cordova et al., 2022) |
| y1_trails_cond3 | Trail Making Task – Condition 3 | comb_adhd | Composite parent+teacher ADHD (Cordova et al., 2022) |

Table S3. Auxiliary variables included in the imputation models.

|  | ABCD | | | | Oregon-ADHD-1000 | |
| --- | --- | --- | --- | --- | --- | --- |
| Family Size | All | White / European | Hispanic / Latino | Black / AA | All | White / European |
| 1 | 8148 | 3768 | 914 | 1062 | 956 | 432 |
| 2 | 1791 | 869 | 105 | 186 | 236 | 87 |
| 3 | 44 | 17 | 2 | 3 | 7 | 1 |
| 5 | 1 | 1 | 0 | 0 | 0 | 0 |

Table S4. Family sizes based on self-reported relationships in both cohorts.

| Cohort/Risk Factor | Effect Estimate | P-value | R^2^ |
| --- | --- | --- | --- |
| ABCD – White/European |  |  |  |
| Family Income | -0.125 (-0.158, -0.092) | 1.05e-13 | *0.036* |
| Family Conflict | 0.192 (0.163, 0.221) | <2e-16 | *0.058* |
| ADHD-PRS | 0.092 (0.064, 0.120) | 6.51e-11 | *0.031* |
| CUD-PRS | 0.041 (0.014, 0.068) | 0.00330 | *0.021* |
| Total R^2^ |  |  | 0.086 |
| ABCD Matched – White/European |  |  |  |
| Family Income | -0.138 (-0.218, -0.058) | 7.98e-4 | *0.048* |
| Family Conflict | 0.244 (0.170, 0.318) | 1.63e-10 | *0.081* |
| ADHD-PRS | 0.190 (0.112, 0.266) | 1.71e-6 | *0.057* |
| Total R^2^ |  |  | 0.136 |
| Oregon-ADHD-1000 – White/European |  |  |  |
| Negative Sentiment | 0.337 (0.266, 0.409) | <2e-16 | *0.139* |
| ADHD-PRS | 0.167 (0.094, 0.240) | 8.48e-6 | *0.060* |
| Total R^2^ |  |  | 0.166 |

Table S5. Results of the **reduced additive-effects models for inattention symptoms*** are shown **for the European-ancestry subgroups** of both the ABCD and Oregon-ADHD-1000 cohorts. The effect estimates (standardized regression coefficients) and p-values are those from the additive model containing all risk factors listed for each cohort. R^2^ values are reported for each individual risk factor (from the univariate main effects models; *italicized*) as well as for the reduced additive model shown (Total R^2^). *Inattention symptoms were measured by the CBCL attention problems T-score and Conners inattention T-score in the two cohorts, respectively.

| Risk Factor | Effect Estimate (P-value) | | | |
| --- | --- | --- | --- | --- |
|  | CBCL ADHD DSM-oriented T-score | CBCL Attention Problems T-score | KSADS Inattention Symptoms | KSADS Hyperactivity Symptoms |
| Family Income | -0.0956 (2.76e-8) | -0.125 (1.05e-13) | -0.0680 (2.60e-5) | -0.0702 (4.41e-5) |
| Family Conflict | 0.201 (<2e-16) | 0.192 (<2e-16) | 0.131 (<2e-16) | 0.138 (<2e-16) |
| ADHD-PRS | 0.0977 (2.15e-12) | 0.092 (6.51e-11) | 0.0511 (2.86e-4) | 0.0454 (0.00147) |
| CUD-PRS | NS | 0.041 (0.00330) | NS | NS |
| ADI | 0.0198 (0.00149) | NS | NS | NS |

Table S6. Comparison of results of the reduced additive-effects models across various symptom measures in the European-ancestry subgroup of ABCD. NS = not significant.

| Risk Factor | Effect Estimate (P-value) | | | | | | |
| --- | --- | --- | --- | --- | --- | --- | --- |
|  | ADHDRS Total Symptoms  T-score | ADHDRS Inatt.  T-score | ADHDRS Hyp.  T-score | Conners Inatt.  T-score | Conners Hyp.  T-score | KSADS Inatt. Symptoms | KSADS Hyp. Symptoms |
| Family Conflict | 0.331  (<2e-16) | 0.36  (<2e-16) | 0.261 (2.19e-9) | 0.337  (<2e-16) | 0.251 (3.81e-9) | 0.336  (2.22e-16) | 0.232 (6.68e-8) |
| ADHD-PRS | 0.192 (2.09e-7) | 0.159 (2.01e-4) | 0.185 (8.18e-6) | 0.167 (8.48e-6) | 0.172 (3.81e-5) | 0.174 (3.97e-5) | 0.195 (3.20e-6) |

Table S7. Comparison of results of the reduced additive-effects models across various symptom measures in the European-ancestry subgroup of the Oregon-ADHD-1000 cohort.

| Cohort/Risk Factor | Effect Estimate | P-value | AUC-ROC |
| --- | --- | --- | --- |
| ABCD Matched – White/European |  |  |  |
| Family Conflict | 0.516 (0.347, 0.685) | 3.67e-9 | *0.658* |
| ADHD-PRS | 0.482 (0.297, 0.668) | 4.31e-7 | *0.636* |
| Total AUC-ROC |  |  | 0.699 |
| Oregon-ADHD-1000 – White/European |  |  |  |
| Negative Sentiment | 0.854 (0.630, 1.077) | 3.54e-13 | *0.740* |
| ADHD-PRS | 0.512 (0.304, 0.721) | 1.79e-6 | *0.683* |
| Total AUC-ROC |  |  | 0.764 |

Table S8. Results of the **reduced additive-effects models for ADHD/non-ADHD status** are shown **for the European-ancestry subgroups** of both the ABCD (N=732) and Oregon-ADHD-1000 (N=599) cohorts. The effect estimates (log of the odds ratio) and p-values are those from the additive model containing all risk factors listed for each cohort. Area under the receiver operating characteristic curve (AUC-ROC) values are reported for each individual risk factor (from the univariate main effects models; *italicized*) as well as for the reduced additive model shown (Total AUC-ROC).

|  |  | ABCD – White/European | | Oregon-ADHD-1000 – White/European | |
| --- | --- | --- | --- | --- | --- |
| Genetic Factor | Exposure | Interaction β | P-value | Interaction β | P-value |
| MDD-PRS | Family Conflict | 0.025 (-0.003, 0.052) | 0.081 | 0.035 (-0.035, 0.104) | 0.332 |
| MDD-PRS | PM2.5 | 0.027 (-0.001,0.054) | 0.057 | 0.006 (-0.076, 0.088) | 0.885 |
| SCZ-PRS | NO_2_ | 0.032 (0.001, 0.062) | 0.040 | 0.047 (-0.013, 0.106) | 0.112 |
| BP-PRS | ADI | -0.026 (-0.056, 0.003) | 0.082 | -0.030 (-0.112, 0.052) | 0.475 |
| BP-PRS | Family Income | 0.036 (0.001, 0.072) | 0.046 | 0.070  (-0.014, 0.153) | 0.105 |
| ASD-PRS | ADI | 0.023 (-0.002, 0.048) | 0.082 | 0.014 (-0.051, 0.079) | 0.673 |
| ANX-PRS | Lead Risk | -0.025 (-0.050, 0.0003) | 0.053 | -0.021 (-0.101, 0.059) | 0.605 |
| ANX-PRS | NO_2_ | -0.028 (-0.054, -0.001) | 0.041 | 0.061 (-0.041, 0.163) | 0.240 |
|  |  | ABCD – Black/African-American | |  |  |
| ASD-PRS | Family Conflict | -0.067 (-0.130, -0.005) | 0.035 | -- | -- |
|  |  | ABCD – Hispanic/Latino | |  |  |
| ADHD-PRS | Lead Risk | -0.049 (-0.100, 0.002) | 0.059 | -- | -- |
| ADHD-PRS | NO_2_ | -0.060 (-0.111, -0.010) | 0.020 | -- | -- |
|  |  | Trans-ancestry meta-analysis | |  |  |
| BP-PRS | ADI | -0.026  (-0.050, -0.002) | 0.031 |  |  |
| ADHD-PRS | Lead Risk | -0.026 (-0.055, 0.003) | 0.080 |  |  |
| ANX-PRS | Lead Risk | -0.024 (-0.045, -0.003) | 0.026 |  |  |
| CUD-PRS | Lead Risk | -0.024 (-0.047, -0.002) | 0.032 |  |  |

Table S9a. **GxE effects on total ADHD symptoms** in the ABCD and Oregon-ADHD-1000 cohorts, **stratified by ancestry subgroup and also meta-analyzed**. Only those interactions with an unadjusted p-value ≤ 0.1 in ABCD are shown, along with corresponding effects in the Oregon-ADHD-1000 cohort.

|  |  | ABCD – All | | Oregon-ADHD-1000 – All | |
| --- | --- | --- | --- | --- | --- |
| Genetic Factor | Exposure | Interaction β | P-value | Interaction β | P-value |
| ADHD-PRS | Family Income | 0.020 (-0.004, 0.044) | 0.099 | 0.029 (-0.028, 0.087) | 0.316 |
| ADHD-PRS | Lead Risk | -0.021 (-0.041, -0.002) | 0.034 | 0.011 (-0.049, 0.071) | 0.719 |
| MDD-PRS | Family Income | 0.019 (-0.003, 0.041) | 0.089 | 0.008 (-0.056, 0.072) | 0.813 |
| MDD-PRS | Family Conflict | 0.022 (0.001, 0.042) | 0.040 | 0.001 (-0.060, 0.062) | 0.977 |
| SCZ-PRS | Family Income | **0.051 (0.028, 0.075)** | **1.41e-5** | 0.012 (-0.049, 0.073) | 0.706 |
| BP-PRS | ADI | -0.029 (-0.051, -0.006) | 0.012 | -0.005 (-0.063, 0.054) | 0.876 |
| BP-PRS | Family Income | **0.049 (0.025, 0.072)** | **4.32e-5** | 0.041 (-0.019, 0.102) | 0.177 |
| BP-PRS | Lead Risk | -0.019 (-0.040, 0.001) | 0.067 | -0.013 (-0.069, 0.044) | 0.665 |
| ASD-PRS | Family Income | 0.028 (0.005, 0.050) | 0.016 | 0.020 (-0.040, 0.079) | 0.520 |
| CUD-PRS | Family Income | **0.048 (0.025, 0.070)** | **2.58e-5** | 0.019 (-0.043, 0.080) | 0.553 |
| CUD-PRS | Lead Risk | -0.024 (-0.045, -0.004) | 0.019 | 0.032 (-0.025, 0.089) | 0.273 |
| ALCDEP-PRS | Family Income | **0.042 (0.018, 0.065)** | **4.64e-4** | -0.009 (-0.072, 0.054) | 0.782 |

Table S9b. **GxE effects on total ADHD symptoms in the full ABCD and Oregon-ADHD-1000 cohorts**. Only those interactions with an unadjusted p-value ≤ 0.1 in ABCD are shown, along with corresponding effects in the Oregon-ADHD-1000 cohort. Those effects in **bold** were considered significant after correcting for multiple comparisons.

|  |  | ABCD – White/European | | Oregon-ADHD-1000 – White/European | |
| --- | --- | --- | --- | --- | --- |
| Genetic Factor | Exposure | Interaction β | P-value | Interaction β | P-value |
| MDD-PRS | Family Conflict | 0.036  (0.007, 0.064) | 0.015 | 0.0118 (-0.059, 0.083) | 0.744 |
| SCZ-PRS | Family Income | 0.049 (0.010, 0.088) | 0.014 | 0.0442 (-0.034, 0.122) | 0.267 |
| SCZ-PRS | NO_2_ | 0.033 (0.002, 0.064) | 0.035 | 0.049 (-0.017, 0.116) | 0.148 |
| BP-PRS | ADI | -0.027 (-0.055, 0.002) | 0.066 | -0.0144 (-0.094, 0.065) | 0.724 |
| BP-PRS | Family Income | 0.054 (0.015,0.090) | 0.005 | 0.0721 (-0.005, 0.150) | 0.069 |
| ANX-PRS | Lead Risk | -0.021 (-0.047, 0.002) | 0.077 | -0.0224 (-0.100, 0.055) | 0.572 |
| ANX-PRS | NO_2_ | -0.027 (-0.054, -0.001) | 0.045 | 0.0782 (-0.026, 0.182) | 0.140 |
| CUD-PRS | Family Conflict | 0.026 (-0.003, 0.055) | 0.078 | 0.0274 (-0.044, 0.098) | 0.451 |
|  |  | ABCD – Black/African-American | |  |  |
| ASD-PRS | Family Conflict | -0.064 (-0.123, -0.004) | 0.036 | -- | -- |
|  |  | ABCD – Hispanic/Latino | |  |  |
| ADHD-PRS | Lead Risk | -0.073 (-0.128, -0.019) | 0.009 | -- | -- |
| ADHD-PRS | NO_2_ | -0.060 (-0.115, -0.005) | 0.033 | -- | -- |
| SCZ-PRS | Lead Risk | -0.055 (-0.116, 0.005) | 0.074 | -- | -- |
| ANX-PRS | PM2.5 | -0.445  (-0.088, -0.001) | 0.047 | -- | -- |
| CUD-PRS | Lead Risk | -0.050 (-0.104, 0.005) | 0.074 | -- | -- |
| CUD-PRS | NO_2_ | -0.045 (-0.091, 0.002) | 0.062 | -- | -- |
| ALCDEP-PRS | Income | 0.054 (-0.001, 0.110) | 0.056 | -- | -- |
|  |  | Trans-ancestry meta-analysis | |  |  |
| BP-PRS | ADI | -0.029 (-0.052, -0.005) | 0.019 |  |  |
| MDD-PRS | Family Conflict | 0.029 (0.004, 0.054) | 0.022 |  |  |
| ANX-PRS | Lead Risk | -0.021 (-0.042, -3e-4) | 0.047 |  |  |
| ANX-PRS | PM2.5 | -0.019 (-0.040; 0.002) | 0.070 |  |  |

Table S10a. **GxE effects on inattention symptoms*** in the ABCD and Oregon-ADHD-1000 cohorts, **stratified by ancestry subgroup and also meta-analyzed**. Only those interactions with an unadjusted p-value ≤ 0.1 in ABCD are shown, along with the corresponding effects in the Oregon-ADHD-1000 cohort. *Inattention symptoms were measured by the CBCL attention problems T-score and Conners inattention T-score in the two cohorts, respectively.

|  |  | ABCD – All | | Oregon-ADHD-1000 – All | |
| --- | --- | --- | --- | --- | --- |
| Genetic Factor | Exposure | Interaction β | P-value | Interaction β | P-value |
| ADHD-PRS | Family Income | 0.027 (0.003, 0.052) | 0.026 | 0.031 (-0.026, 0.087 | 0.287 |
| ADHD-PRS | Lead Risk | -0.019 (-0.039, 0.001) | 0.068 | 0.026 (-0.033, 0.085) | 0.393 |
| MDD-PRS | Family Income | 0.022 (-0.0003, 0.045) | 0.054 | 0.006 (-0.053, 0.064) | 0.844 |
| MDD-PRS | Family Conflict | 0.027 (0.005, 0.050) | 0.018 | 0.001 (-0.056, 0.058) | 0.981 |
| SCZ-PRS | ADI | -0.021 (-0.046, 0.004) | 0.095 | -0.007 (-0.066, 0.053) | 0.820 |
| SCZ-PRS | Family Income | **0.062 (0.038, 0.086)** | **4.01e-7** | 0.011 (-0.044, 0.065) | 0.704 |
| BP-PRS | ADI | -0.035 (-0.058, -0.013) | 0.002 | -0.002 (-0.061, 0.057) | 0.946 |
| BP-PRS | Family Income | **0.061 (0.036, 0.085)** | **1.02e-6** | 0.038 (-0.016, 0.091) | 0.172 |
| ASD-PRS | ADI | -0.018 (-0.039, 0.003) | 0.097 | 0.010 (-0.049, 0.069) | 0.739 |
| ASD-PRS | Family Income | 0.037 (0.015, 0.060) | 0.001 | 0.031 (-0.028, 0.089) | 0.302 |
| CUD-PRS | Family Income | **0.058 (0.035, 0.080)** | **7.09e-7** | 0.011 (-0.049, 0.070) | 0.728 |
| CUD-PRS | Family Conflict | 0.018 (-0.003, 0.040) | 0.099 | -0.001 (-0.060, 0.058) | 0.979 |
| CUD-PRS | Lead Risk | -0.021 (-0.041, -0.0004) | 0.045 | 0.046 (-0.014, 0.105) | 0.132 |
| AUDIT-PRS | Family Conflict | 0.019 (-0.002, 0.040) | 0.079 | 0.009 (-0.049, 0.066) | 0.770 |
| ALCDEP-PRS | Family Income | **0.047 (0.024, 0.070)** | **6.95e-5** | -0.011 (-0.071, 0.050) | 0.727 |

Table S10b. **GxE effects on inattention symptoms* in the full ABCD and Oregon-ADHD-1000 cohorts**. Only those interactions with an unadjusted p-value ≤ 0.1 in ABCD are shown, along with corresponding effects in the Oregon-ADHD-1000 cohort. **Bold** indicates significant after correction. *Inattention symptoms were measured by the CBCL attention problems T-score and Conners inattention T-score in the two cohorts, respectively.

|  |  | ABCD – White/European | | Oregon-ADHD-1000 – White/European | |
| --- | --- | --- | --- | --- | --- |
| Genetic Factor | Exposure | Interaction β | P-value | Interaction β | P-value |
| ADHD-PRS | Family Conflict | -0.190 (-0.364, -0.015) | 0.033 | 0.112 (-0.142, 0.366) | 0.389 |
| ADHD-PRS | Lead Risk | -0.191 (-0.356, -0.026) | 0.024 | 0.114 (-0.071, 0.300) | 0.228 |
| SCZ-PRS | ADI | -0.176 (-0.364, 0.012) | 0.068 | -0.081 (-0.258, 0.096) | 0.372 |
| SCZ-PRS | Family Conflict | -0.187 (-0.364, -0.010) | 0.039 | 0.116 (-0.129, 0.362) | 0.354 |
| BP-PRS | ADI | -0.191 (-0.394, 0.013) | 0.067 | 0.008 (-0.167, 0.183) | 0.930 |
| BP-PRS | Family Income | 0.144 (-0.007, 0.294) | 0.062 | 0.040 (-0.131, 0.211) | 0.645 |
| BP-PRS | NO_2_ | 0.174 (0.008, 0.339) | 0.040 | -0.127 (-0.337, 0.083) | 0.235 |
| AUDIT-PRS | NO_2_ | -0.136 (-0.290, 0.017) | 0.081 | -0.100 (-0.302, 0.102) | 0.333 |
| ALCDEP-PRS | PM2.5 | 0.139 (-0.013, 0.291) | 0.074 | 0.099 (-0.091, 0.290) | 0.308 |

Table S11a. **GxE effects on ADHD/non-ADHD status in the European-ancestry subgroups of the ABCD matched case-control (N=732) and Oregon-ADHD-1000 cohorts (N=599)**. Only those interactions with an unadjusted p-value ≤ 0.1 in ABCD are shown, along with corresponding effects in the Oregon-ADHD-1000 cohort. Those effects in **bold** were considered significant after correcting for multiple comparisons.

|  |  | ABCD – All | | Oregon-ADHD-1000 – All | |
| --- | --- | --- | --- | --- | --- |
| Genetic Factor | Exposure | Interaction β | P-value | Interaction β | P-value |
| ADHD-PRS | ADI | -0.170 (-0.311, -0.029) | 0.019 | -0.066 (-0.230, 0.098) | 0.433 |
| ADHD-PRS | Family Income | 0.171 (0.053, 0.289) | 0.005 | 0.055 (-0.108, 0.217) | 0.510 |
| ADHD-PRS | Lead Risk | -0.137 (-0.254, -0.019) | 0.023 | 0.083 (-0.073, 0.239) | 0.299 |
| MDD-PRS | Family Income | 0.110 (-0.004, 0.223) | 0.059 | 0.021 (-0.132, 0.175) | 0.786 |
| SCZ-PRS | ADI | -0.220 (-0.367, -0.074) | 0.003 | -0.012 (-0.189, 0.164) | 0.890 |
| SCZ-PRS | Family Income | **0.215 (0.104, 0.325)** | **1.41e-4** | -0.024 (-0.173, 0124) | 0.748 |
| BP-PRS | ADI | **-0.235 (-0.374, -0.097)** | **8.87e-4** | 0.004 (-0.149, 0.158) | 0.958 |
| BP-PRS | Family Income | 0.196 (0.080, 0.312) | 9.63e-4 | 0.039 (-0.107, 0.186) | 0.597 |
| ASD-PRS | ADI | -0.152 (-0.286, -0.019) | 0.025 | -0.040 (-0.208, 0.127) | 0.636 |
| ASD-PRS | Family Income | 0.173 (0.062, 0.284) | 0.002 | 0.017 (-0.145, 0.179) | 0.841 |
| CUD-PRS | ADI | -0.194 (-0.340, -0.048) | 0.009 | 0.015 (-0.143, 0.172) | 0.854 |
| CUD-PRS | Family Income | **0.222 (0.103, 0.341)** | **2.58e-4** | 0.015 (-0.142, 0.172) | 0.850 |
| ALCDEP-PRS | ADI | -0.158 (-0.277, -0.038) | 0.010 | 0.028 (-0.122, 0.177) | 0.715 |
| ALCDEP-PRS | Family Income | 0.203 (0.081, 0.326) | 0.001 | -0.033 (-0.186, 0.121) | 0.675 |

Table S11b. **GxE effects on ADHD/non-ADHD status in the full ABCD and Oregon-ADHD-1000 cohorts**. Only those interactions with an unadjusted p-value ≤ 0.1 in ABCD are shown, along with corresponding effects in the Oregon-ADHD-1000 cohort. Those effects in **bold** were considered significant after correcting for multiple comparisons.


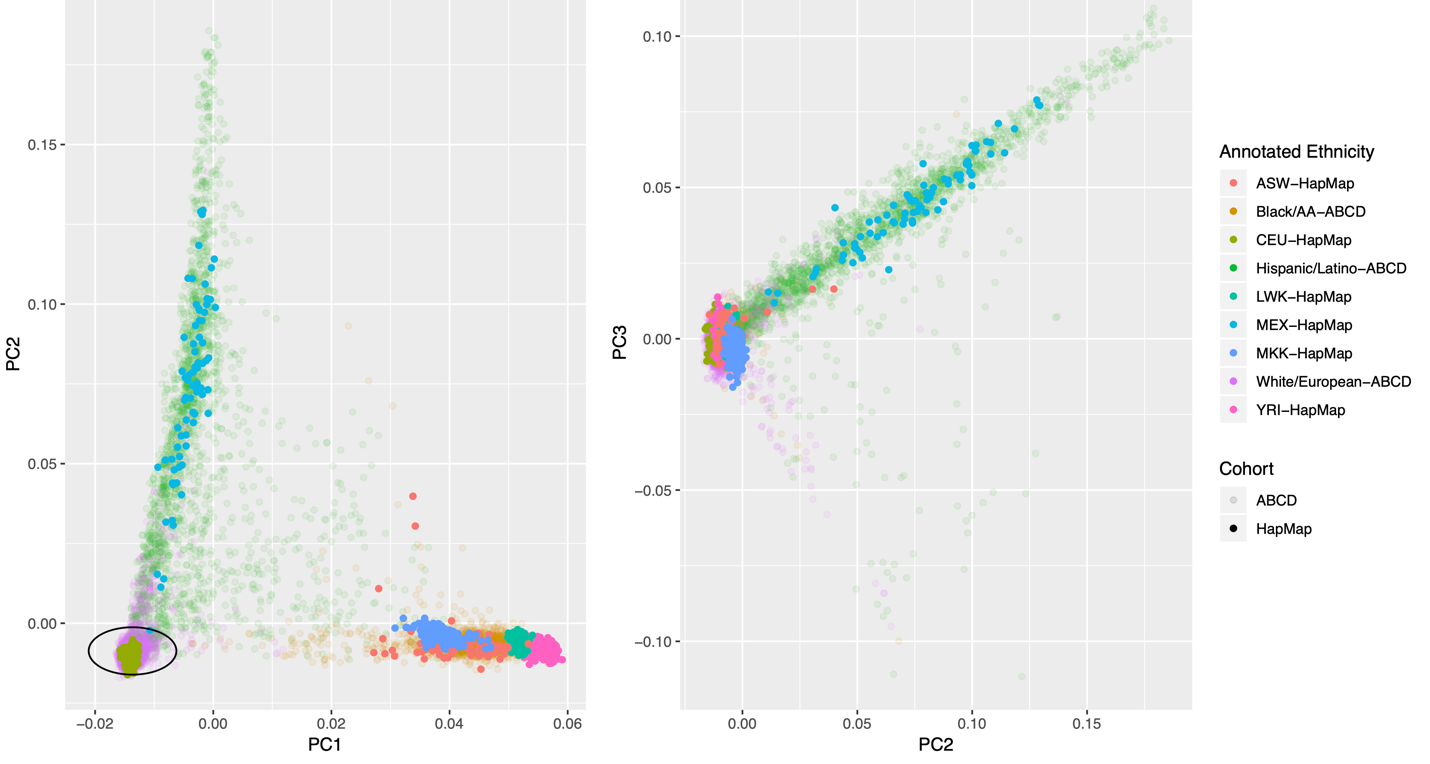


Figure S1. The first three genomic principal components for the three well-represented ancestry subgroups in the ABCD cohort, along with relevant HapMap populations. The black circle in the left plot (PC1 vs. PC2) indicates the criteria used for the homogeneous European-ancestry subgroup (light purple), which aligns well with the CEU (Utah residents with Northern and Western European ancestry from the CEPH collection) HapMap population (left plot). The Hispanic/Latino subgroup (light green), though demonstrating a large amount of variation, aligns well with the MEX (Mexican ancestry in Los Angeles, California) HapMap population (left and right plots). Finally, the Black/African-American subgroup of ABCD (light orange) aligns well with HapMap populations with African ancestry: ASW (African ancestry in Southwest USA), YRI (Yoruba in Ibadan, Nigeria), MKK (Maasai in Kinyawa, Kenya), and LWK (Luhya in Webuye, Kenya) (left plot).


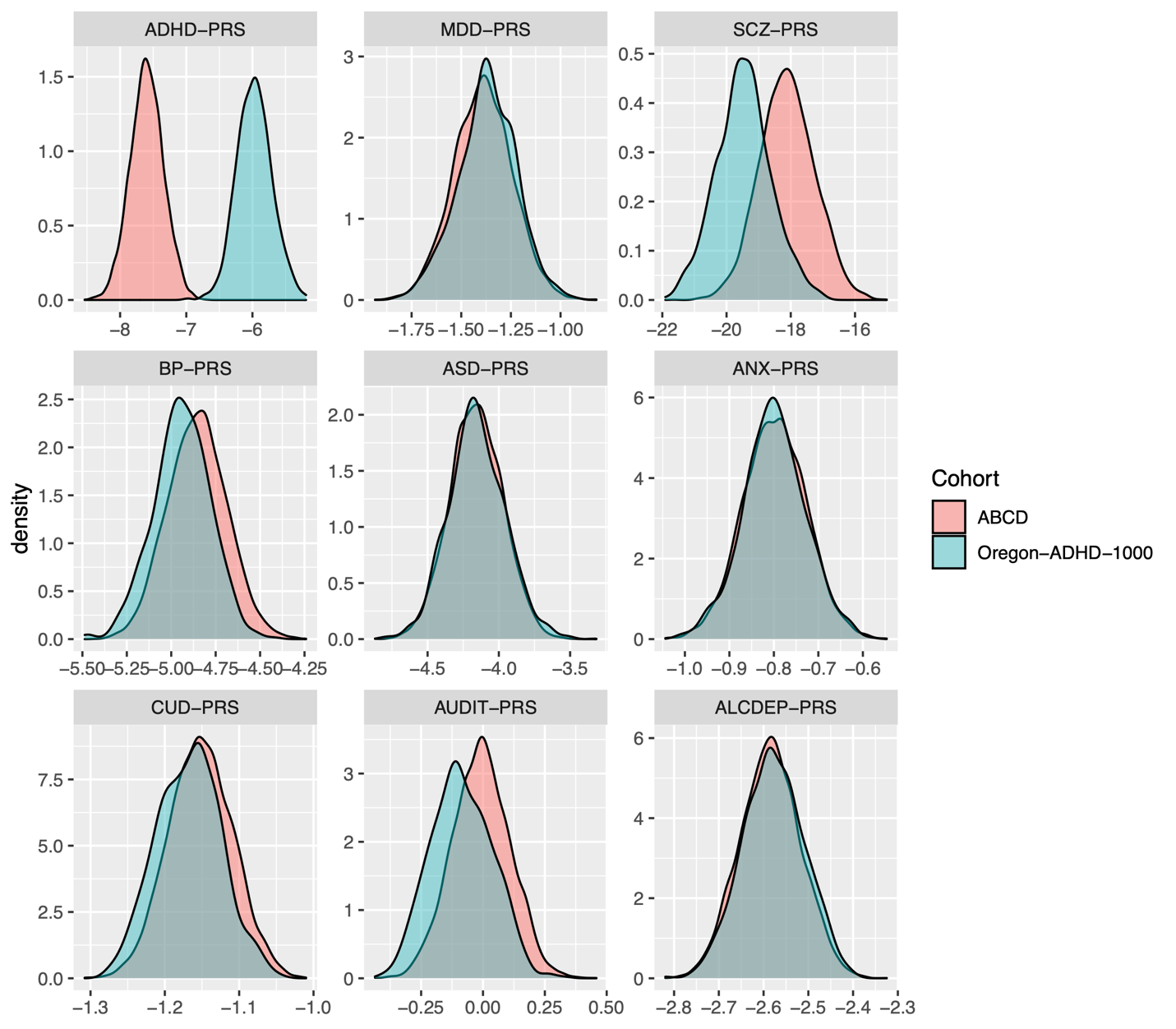


Figure S2. Distributions of all PRS for the European-ancestry subgroups of both cohorts. The raw scores (not centered or scaled) are shown, so that differences between the cohorts can be seen.


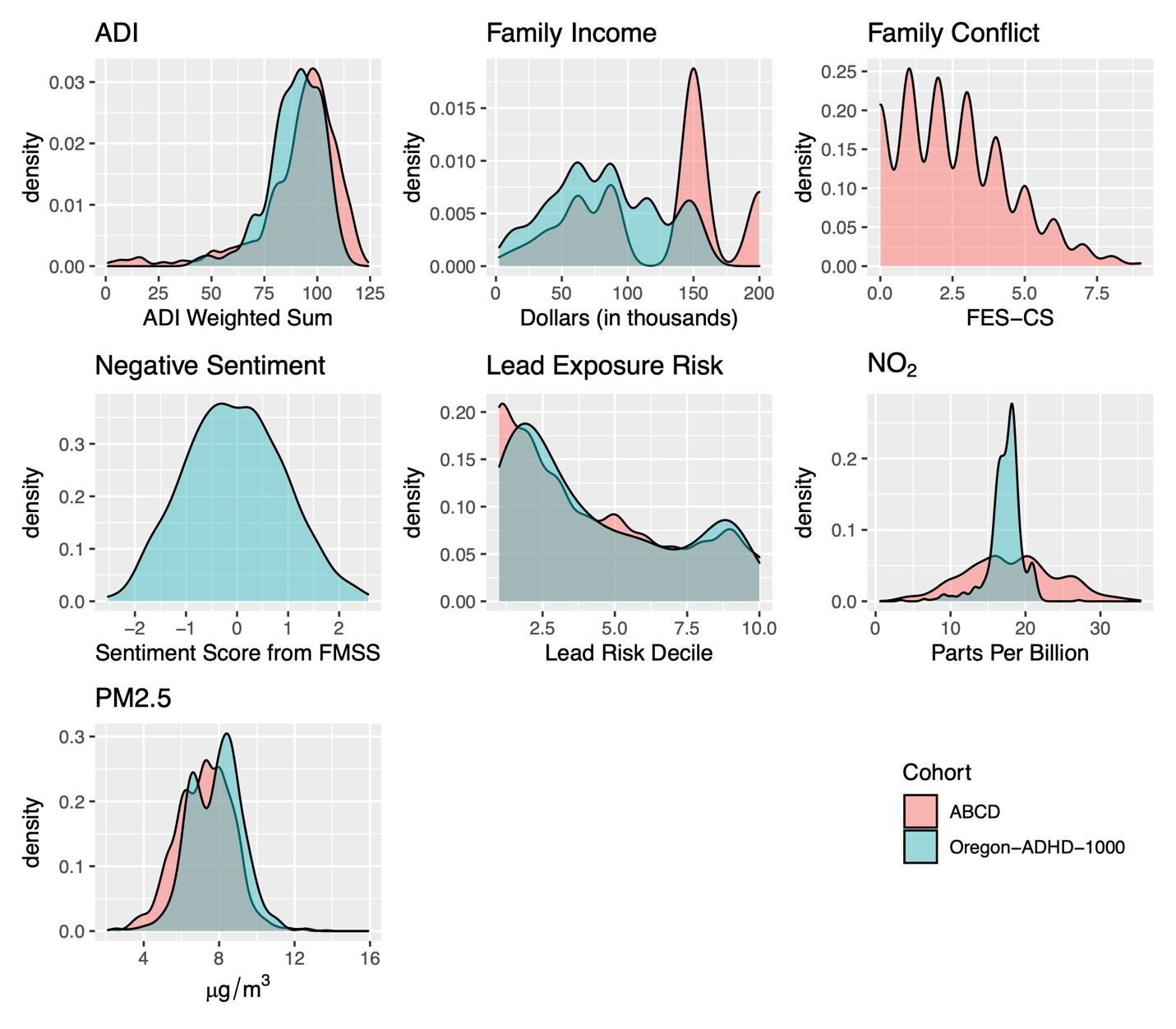


Figure S3. Distributions of all environmental exposures for the European-ancestry subgroups of both cohorts. The raw measures (not centered or scaled) are shown, so that differences between the cohorts can be seen. FES-CS = Conflict Subscale of the Family Environment Scale, FMSS = Five Minute Speech Sample.


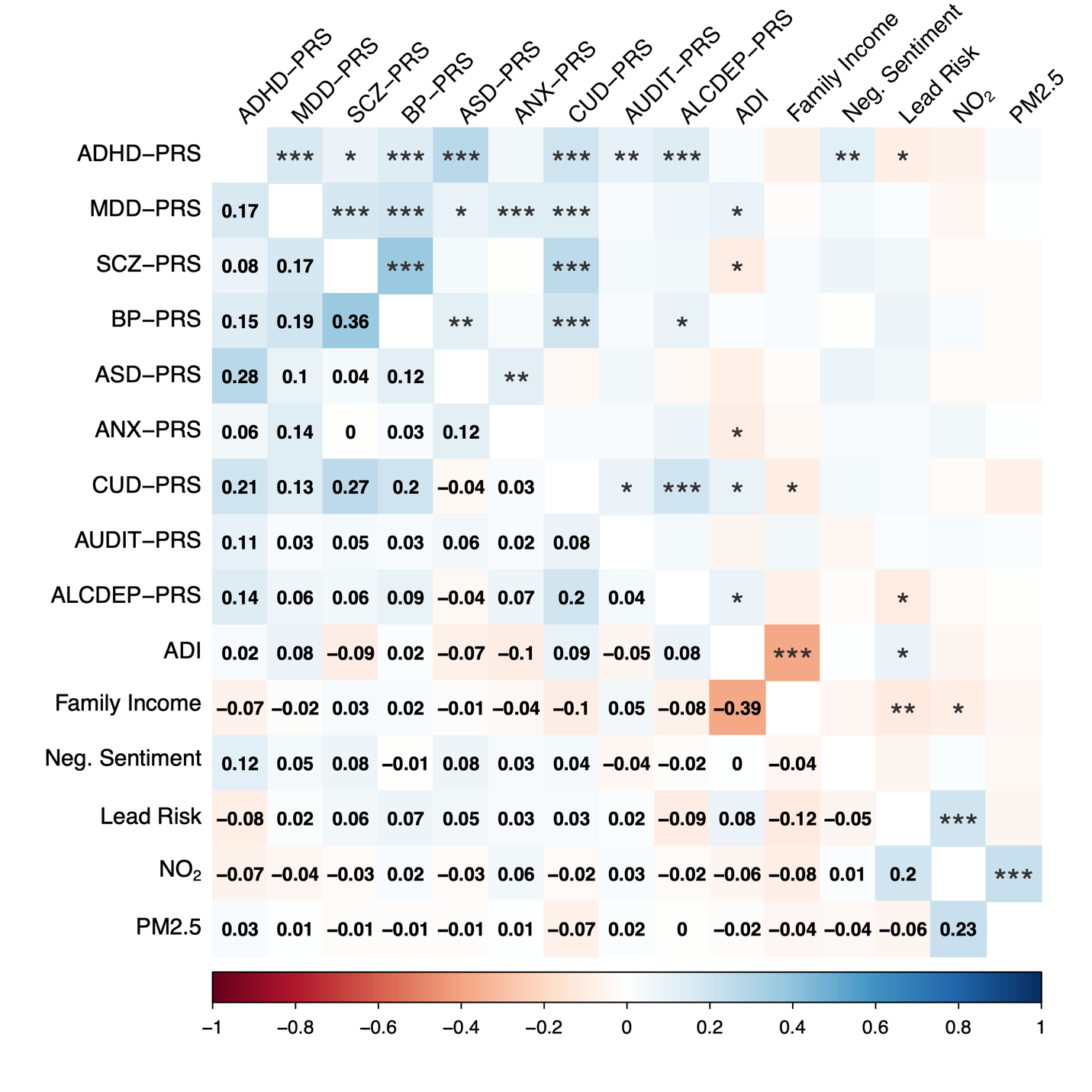


Figure S4. Correlation matrix of PRS and exposures for the European-ancestry subgroup of the Oregon-ADHD-1000 cohort. Correlation coefficients are shown in the lower triangle and significance codes in the upper triangle, with *, **, and *** representing p<0.05, p<0.01, and p<0.001, respectively. ADI = area deprivation index, NO_2_ = nitrogen dioxide, PM2.5 = fine particulate matter, PRS = polygenic risk score, ADHD = attention-deficit/hyperactivity disorder, MDD = major depressive disorder, SCZ = schizophrenia, BP = bipolar disorder, ASD = autism spectrum disorder, ANX = anxiety, CUD = cannabis use disorder, AUDIT = alcohol use disorder, ALCDEP = alcohol dependency.


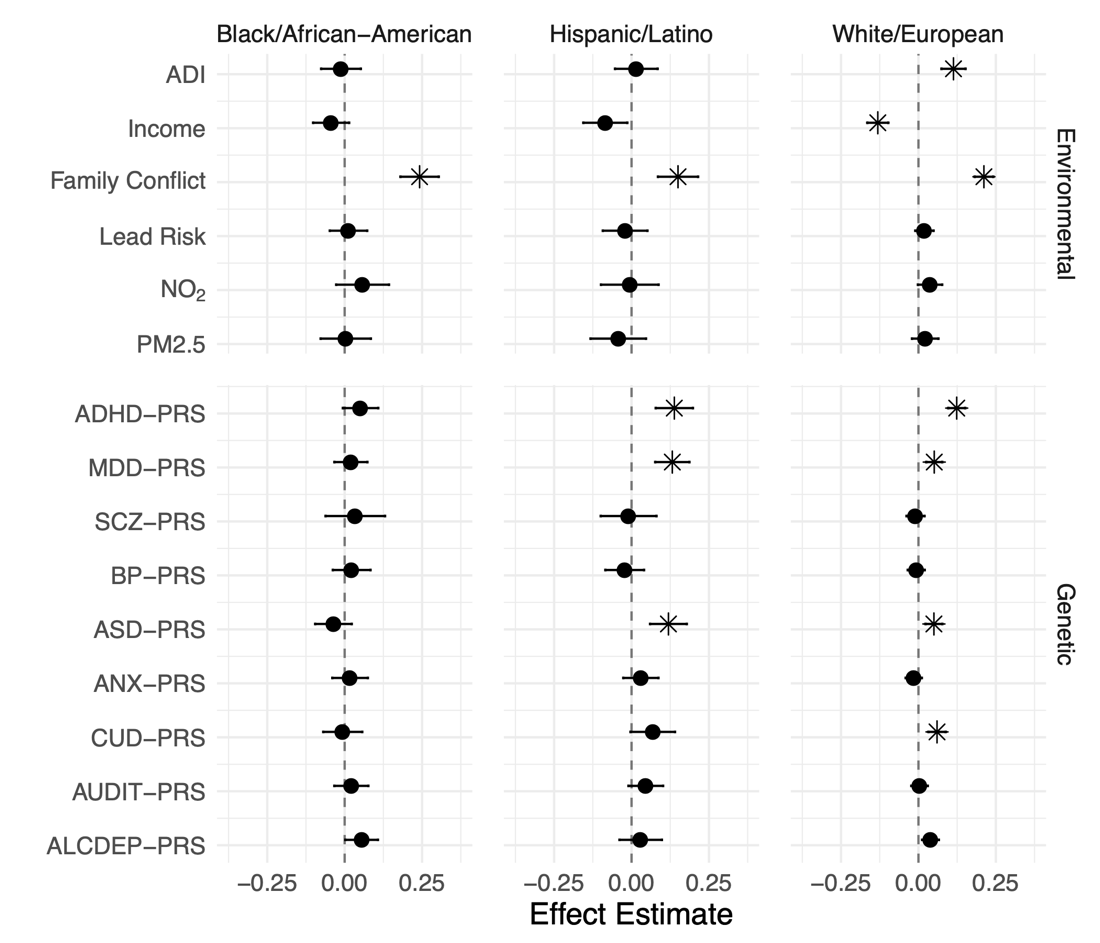


Figure S5. **Univariate main effects on total ADHD symptoms** across **ancestry subgroups in ABCD**. Effect estimates indicated with an asterisk (*) a statistically significant after adjusting for multiple comparisons.


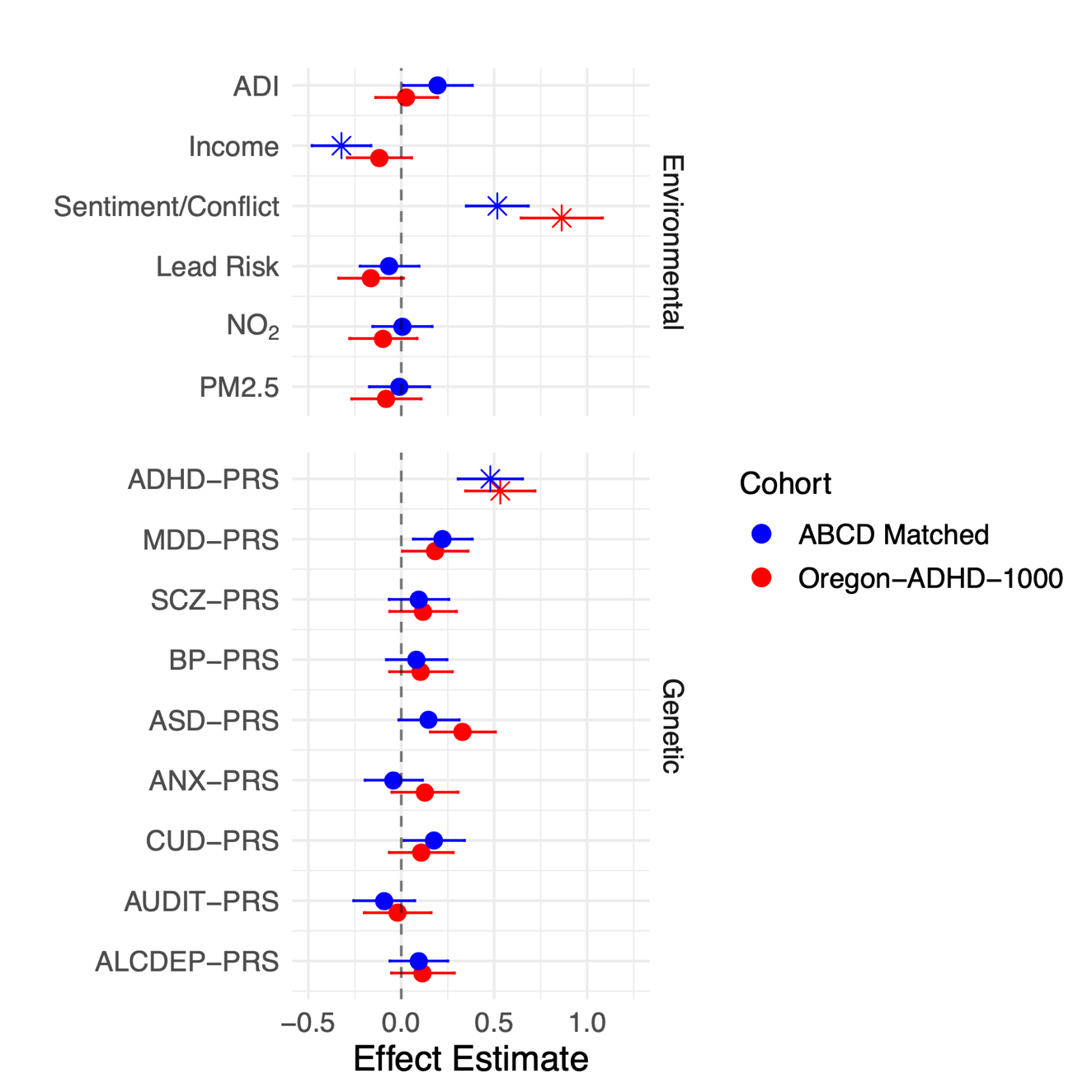


Figure S6. **Univariate main effects** (log of odds ratio) on **ADHD/non-ADHD status in the European-ancestry subgroups of the Oregon-ADHD-1000 and ABCD matched case-control cohorts**. Point estimates indicated with an asterisk (*) are either (a) statistically significant after multiple-testing correction in ABCD, or (b) significantly replicated in the Oregon-ADHD-1000 (p<0.05).


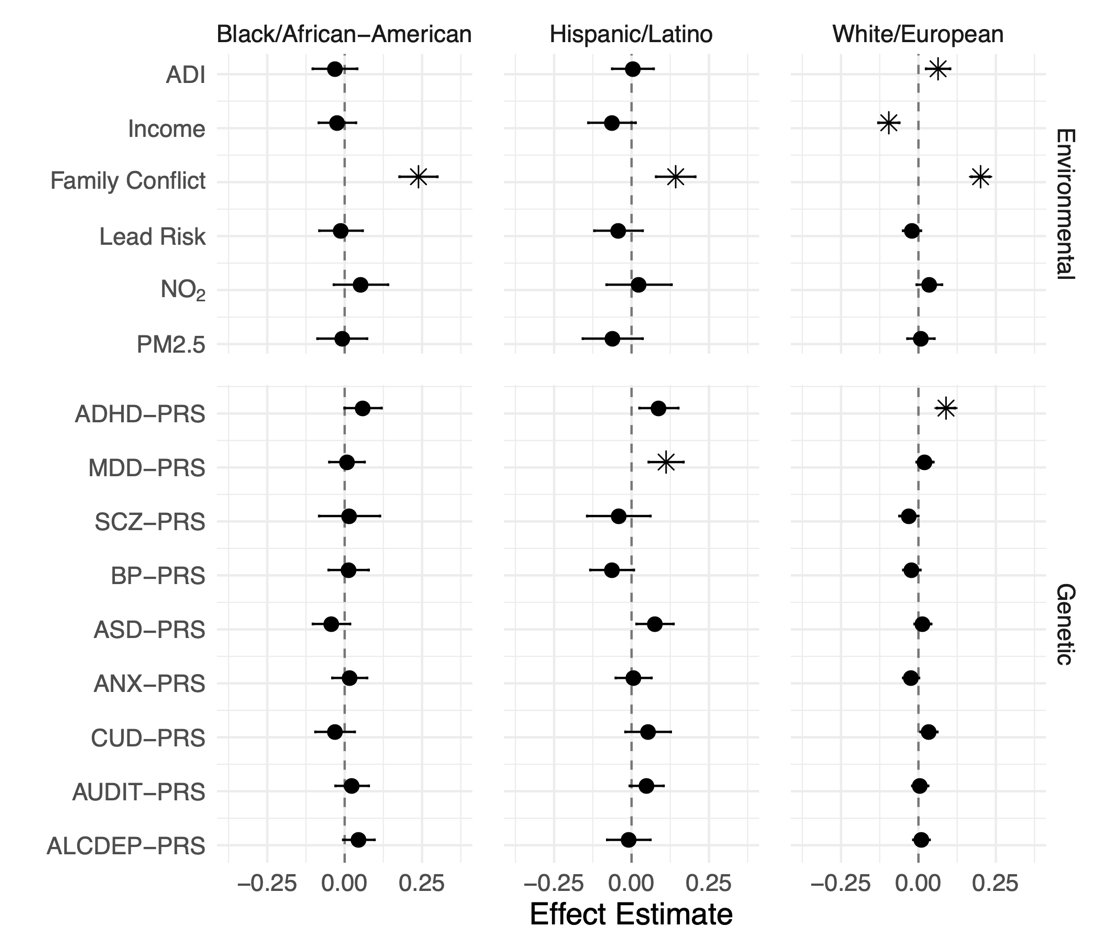


Figure S7. **Results of additive-effects models for total ADHD symptoms**, stratified by ancestry subgroups in ABCD. Effect estimates are from models that include all PRS and environmental exposures together in the same model (i.e., estimates for individual factors after adjusting for all others). Effect estimates indicated with an asterisk (*) are statistically significant after adjusting for multiple comparisons.


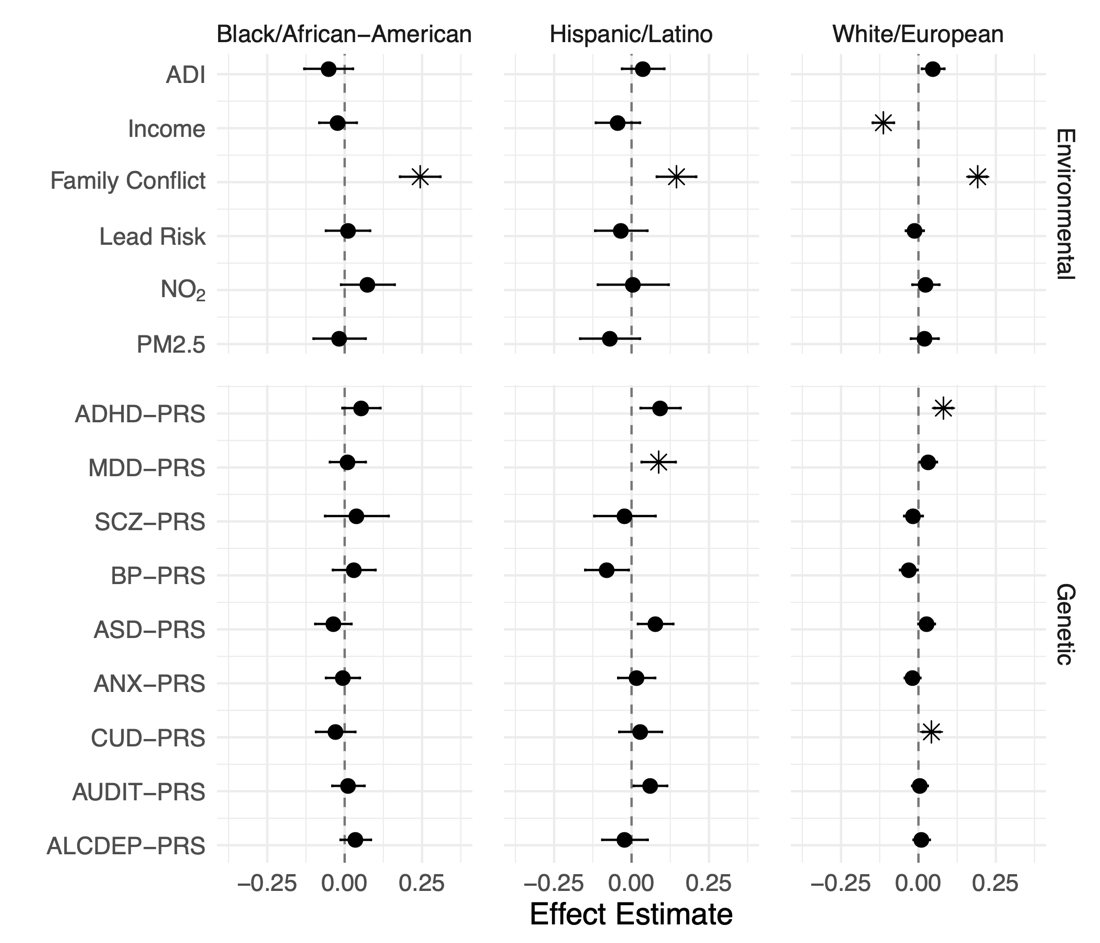


Figure S8. **Results of additive-effects models for inattention symptoms***, stratified by ancestry subgroups in ABCD. Effect estimates are from models that include all PRS and environmental exposures together in the same model (i.e., estimates for individual factors after adjusting for all others). Effect estimates indicated with an asterisk (*) are statistically significant after adjusting for multiple comparisons. *Inattention symptoms were measured by the CBCL attention problems T-score and Conners inattention T-score in the two cohorts, respectively.


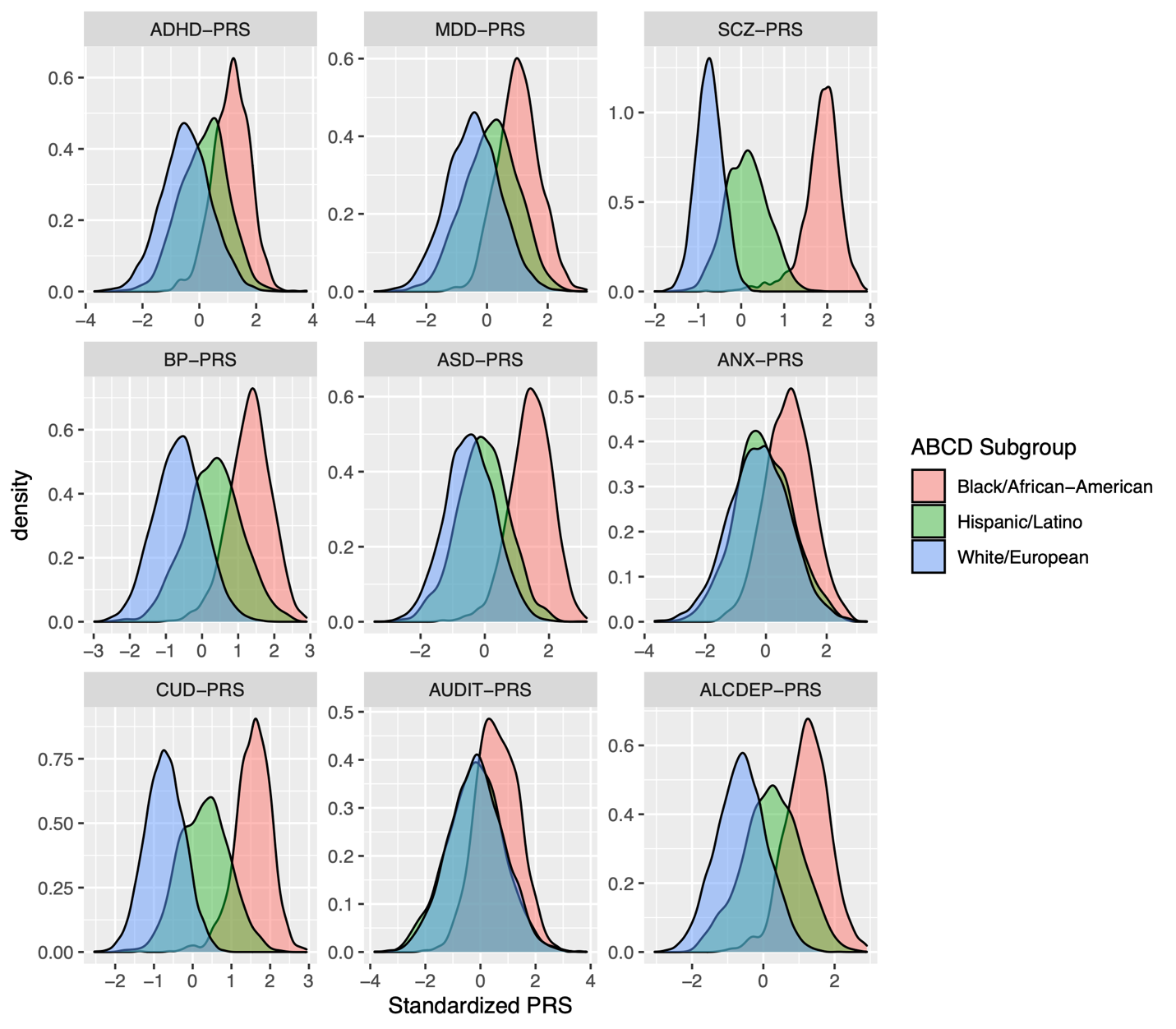


Figure S9. Distributions of all PRS in the ABCD cohort, stratified by ancestry subgroup. PRS are standardized (mean = 0, standard deviation = 1)


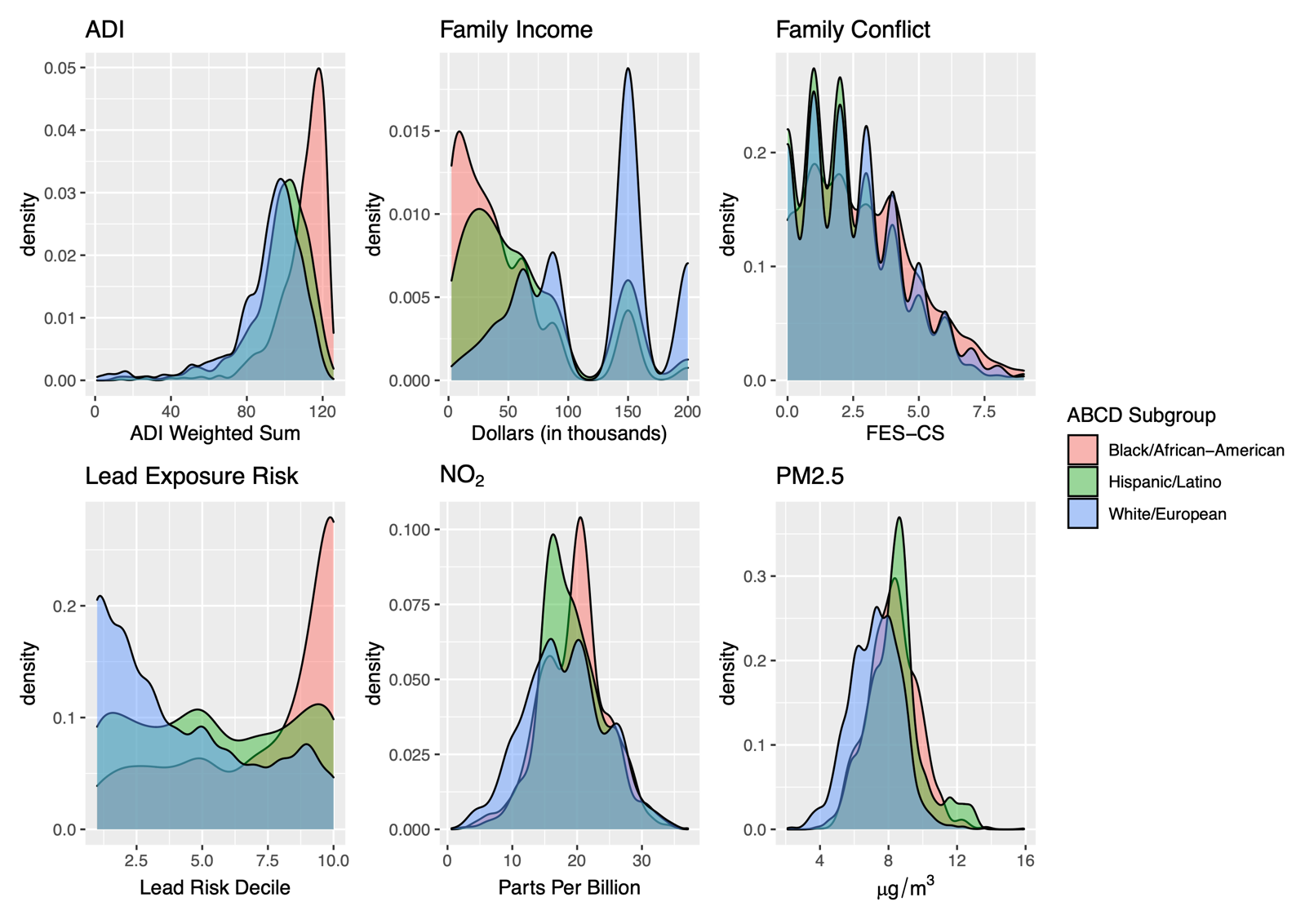


Figure S10. Distributions of all environmental exposures in the ABCD cohort, stratified by ancestry subgroup. The raw measures (not centered or scaled) are shown. FES-CS = Conflict Subscale of the Family Environment Scale.

References for Supplemental Materials:

Cordova, M. M., Antovich, D. M., Ryabinin, P., Neighbor, C., Mooney, M. A., Dieckmann, N. F., Miranda-Dominguez, O., Nagel, B. J., Fair, D. A., & Nigg, J. T. (2022). Attention-Deficit/Hyperactivity Disorder: Restricted Phenotypes Prevalence, Comorbidity, and Polygenic Risk Sensitivity in the ABCD Baseline Cohort. *Journal of the American Academy of Child & Adolescent Psychiatry*. https://doi.org/10.1016/j.jaac.2022.03.030

Devlin, J., Chang, M.-W., Lee, K., & Toutanova, K. (2019). *BERT: Pre-training of Deep Bidirectional Transformers for Language Understanding* (arXiv:1810.04805). arXiv. https://doi.org/10.48550/arXiv.1810.04805

Heeringa, S. G., & Berglund, P. A. (2020). *A Guide for Population-based Analysis of the Adolescent Brain Cognitive Development (ABCD) Study Baseline Data* (p. 2020.02.10.942011). bioRxiv. https://doi.org/10.1101/2020.02.10.942011

Ho, D., Imai, K., King, G., & Stuart, E. A. (2011). MatchIt: Nonparametric Preprocessing for Parametric Causal Inference. *Journal of Statistical Software*, *42*, 1–28. https://doi.org/10.18637/jss.v042.i08

HuggingFace. (2016). *Hugging Face: The AI community building the future*. https://huggingface.co/

McCaw, Z. R., Lane, J. M., Saxena, R., Redline, S., & Lin, X. (2020). Operating characteristics of the rank-based inverse normal transformation for quantitative trait analysis in genome-wide association studies. *Biometrics*, *76*(4), 1262–1272. https://doi.org/10.1111/biom.13214

Nigg, J. T., Gustafsson, H. C., Karalunas, S. L., Ryabinin, P., McWeeney, S. K., Faraone, S. V., Mooney, M. A., Fair, D. A., & Wilmot, B. (2018). Working Memory and Vigilance as Multivariate Endophenotypes Related to Common Genetic Risk for Attention-Deficit/Hyperactivity Disorder. *Journal of the American Academy of Child and Adolescent Psychiatry*, *57*(3), 175–182. https://doi.org/10.1016/j.jaac.2017.12.013

Selah, K., Gustafsson, H. C., Sims, Z., Peris, T. S., Karalunas, S. L., & Nigg, J. T. (under review). Computationally Derived Parent Emotional Sentiment Scores as a Predictor for Change in Child ADHD and ODD. *Journal of Child Psychology and Psychiatry*.

Socher, R., Perelygin, A., Wu, J., Chuang, J., Manning, C. D., Ng, A., & Potts, C. (2013). Recursive Deep Models for Semantic Compositionality Over a Sentiment Treebank. *Proceedings of the 2013 Conference on Empirical Methods in Natural Language Processing*, 1631–1642. https://aclanthology.org/D13-1170

Thompson, W. K., Barch, D. M., Bjork, J. M., Gonzalez, R., Nagel, B. J., Nixon, S. J., & Luciana, M. (2019). The structure of cognition in 9 and 10 year-old children and associations with problem behaviors: Findings from the ABCD study’s baseline neurocognitive battery. *Developmental Cognitive Neuroscience*, *36*, 100606. https://doi.org/10.1016/j.dcn.2018.12.004
